# Supplementary material for: Low-density lipoprotein cholesterol and all-cause mortality: findings from the China health and retirement longitudinal study
Source: BMJ Open. 2020 Aug 16;10(8):e036976. doi: 10.1136/bmjopen-2020-036976 (PMC7430481; doi:10.1136/bmjopen-2020-036976)
Supplement: Supplementary data [file bmjopen-2020-036976supp001.pdf]

Supplementary materials

Low-density lipoprotein cholesterol and all-cause mortality: findings from the China Health and Retirement Longitudinal Study

Liang Zhou [1], Ying Wu [2], Shaobo Yu [3], Yueping Shen [4] and Chaofu Ke [4]

[1] Liyang Center for Disease Control and Prevention, 55 Nanhuan Road, Liyang 213371, P. R. China.

[2] State Key Laboratory of Organ Failure Research, Department of Biostatistics, Guangdong Provincial Key Laboratory of Tropical Disease Research, School of Public Health, Southern Medical University, Guangzhou 510515, China.

[3] Medical College of Soochow University, Suzhou 215123, P. R. China.

[4] Department of Epidemiology and Biostatistics, School of Public Health, Medical College of Soochow University, 199 Renai Road, Suzhou 215123, P. R. China.

Supplementary Table S1 Associations between all-cause mortality and LDL-C[#]

	Total	Deaths (%)	Unadjusted		Adjusted[*]	
			HR (95%CI)	*P* value	HR (95%CI)	*P* value
Men						
Q1	866	75 (8.66)	1	-	1	-
Q2	1008	67(6.65)	0.750(0.539~1.042)	0.0865	0.863(0.551~1.351)	0.5180
Q3	991	57(5.75)	0.653(0.463~0.922)	0.0155	0.833(0.531~1.307)	0.4261
Q4	1004	47(4.68)	0.531(0.368~0.764)	0.0007	0.643(0.399~1.036)	0.0698
Q5	987	46(4.66)	0.524(0.363~0.756)	0.0006	0.861(0.533~1.390)	0.5406
Women						
Q1	1025	46 (4.49)	1	-	1	-
Q2	1102	49(4.45)	0.998(0.667~1.492)	0.9920	1.231(0.735~2.062)	0.4290
Q3	1096	29(2.65)	0.587(0.369~0.935)	0.0248	0.619(0.340~1.127)	0.1167
Q4	1111	41(3.69)	0.822(0.540~1.253)	0.3628	0.840(0.480~1.472)	0.5430
Q5	1106	48(4.34)	0.962(0.642~1.441)	0.8511	0.939(0.548~1.609)	0.8180

[#]Participants with LDL-C < 50 mg/dL were excluded.

[*]Adjusted for age, smoking, drinking, BMI, living alone, household income, educational level, rural residence, ADL disability, hand grip strength, HDL-C, triglyceride, hemoglobin, hypertension, HBS/diabetes, history of stroke, cancer, heart disease, lung disease, liver disease, kidney disease, digestive disease, asthma, arthritis, psychological problem and memory problem.

BMJ Publishing Group Limited (BMJ) disclaims all liability and responsibility arising from any reliance placed on this supplemental material which has been supplied by the author(s)

Supplementary Table S2 Analyses of interactions between LDL-C and potential risk factors

ID	Interaction term	*P* value in men	*P* value in women
1	LDL-C*age	*0.7323*	*0.0931*
2	LDL-C*obesity (BMI⩾28)	*0.4033*	*0.4825*
3	LDL-C*rural residence	*0.1426*	*0.8102*
4	LDL-C*ADL disability	*0.4108*	*0.5052*
5	LDL-C*smoking	*0.6150*	*0.0498*
6	LDL-C*drinking	*0.8680*	*0.4018*
7	LDL-C*hypertension	*0.7685*	*0.8919*
8	LDL-C*diabetes	0.7151	0.1260
9	LDL-C*heart disease	0.8480	0.9988
10	LDL-C*stroke	0.1101	0.3961
11	LDL-C*cancer	0.6451	0.6695
12	LDL-C*lung disease	0.6657	0.4847
13	LDL-C*memory disease	0.6225	0.4887
14	LDL-C*kidney disease	0.1251	0.1876
15	LDL-C*arthritis	0.4297	0.1671
16	LDL-C*asthma	0.4187	0.4779
17	LDL-C*liver disease	0.6929	0.7013
18	LDL-C*digestive disease	0.1048	0.4019
19	LDL-C*psychological disease	0.4605	0.3731

Supplementary Table S3 Associations between all-cause mortality and LDL-C#

	Total	Deaths (%)	Unadjusted		Adjusted*	
			HR (95%CI)	*P* value	HR (95%CI)	*P* value
Men						
Q1	972	69 (7.10)	1	-	1	-
Q2	999	58 (5.81)	0.806(0.568~1.142)	0.2251	0.921(0.582~1.457)	0.7236
Q3	979	45 (4.60)	0.642(0.441~0.934)	0.0205	0.762(0.472~1.231)	0.2668
Q4	992	35 (3.53)	0.491(0.327~0.737)	0.0006	0.566(0.337~0.951)	0.0315
Q5	982	41 (4.18)	0.579(0.394~0.853)	0.0056	0.841(0.510~1.386)	0.4970
Women						
Q1	1106	44 (3.98)	1	-	1	-
Q2	1094	41 (3.75)	0.951(0.622~1.456)	0.8177	1.171(0.683~2.005)	0.5660
Q3	1094	27 (2.47)	0.622(0.385~1.005)	0.0523	0.648(0.350~1.198)	0.1664
Q4	1105	35 (3.17)	0.799(0.513~1.246)	0.3222	0.815(0.454~1.462)	0.4924
Q5	1099	41 (3.73)	0.934(0.610~1.430)	0.7538	0.910(0.519~1.596)	0.7424

#Participants who died during the first year were excluded.

*Adjusted for age, smoking, drinking, BMI, living alone, household income, educational level, rural residence, ADL disability, hand grip strength, HDL-C, triglyceride, hemoglobin, hypertension, HBS/diabetes, history of stroke, cancer, heart disease, lung disease, liver disease, kidney disease, digestive disease, asthma, arthritis, psychological problem and memory problem.

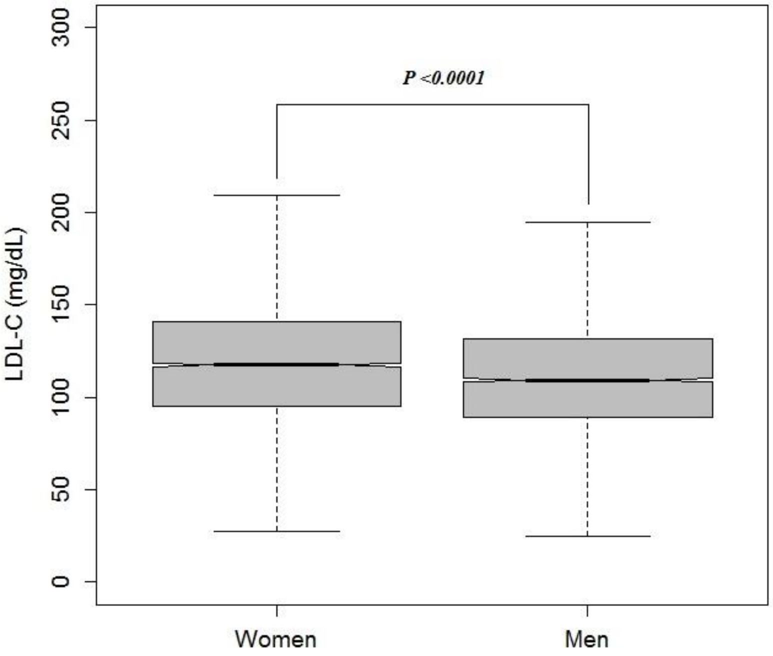

Supplementary Figure S1 The box-plot of plasma LDL-C levels in middle-aged and elderly Chinese men and women.

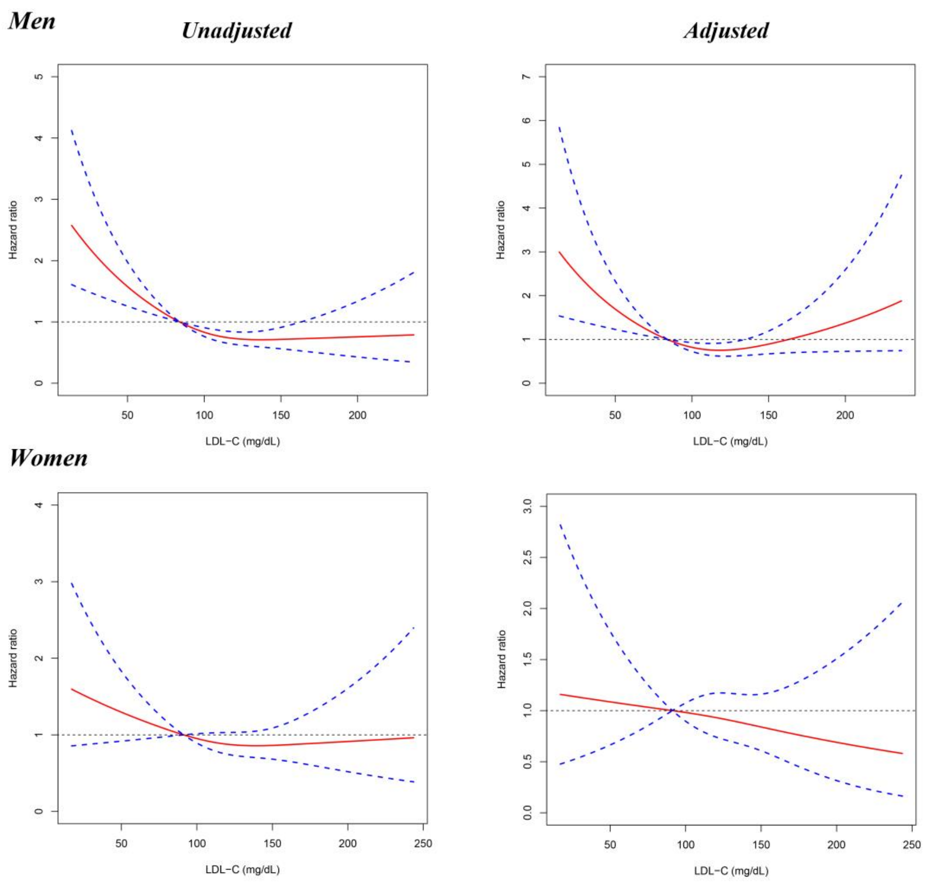

Supplementary Figure S2 Results from restricted cubic splines for the association between LDL-C and 4-year all-cause mortality in men and women (excluding participants who died during the first year). The multivariable models were adjusted for age, smoking, drinking, BMI, living alone, household income, educational level, rural residence, ADL disability, hand grip strength, HDL-C, triglyceride, hemoglobin, hypertension, HBS/diabetes, history of stroke, cancer, heart disease, lung disease, liver disease, kidney disease, digestive disease, asthma, arthritis, psychological problem and memory problem.
