# Supplementary material for: Low-density lipoprotein cholesterol and all-cause mortality: findings from the China health and retirement longitudinal study
Source: BMJ Open. 2020 Aug 16;10(8):e036976. doi: 10.1136/bmjopen-2020-036976 (PMC7430481; doi:10.1136/bmjopen-2020-036976)
Supplement: Author's manuscript [file bmjopen-2020-036976.draft_revisions.pdf]

BMJ Open is committed to open peer review. As part of this commitment we make the peer review history of every article we publish publicly available.

When an article is published we post the peer reviewers' comments and the authors' responses online. We also post the versions of the paper that were used during peer review. These are the versions that the peer review comments apply to.

The versions of the paper that follow are the versions that were submitted during the peer review process. They are not the versions of record or the final published versions. They should not be cited or distributed as the published version of this manuscript.

BMJ Open is an open access journal and the full, final, typeset and author-corrected version of record of the manuscript is available on our site with no access controls, subscription charges or pay-per-view fees (http://bmjopen.bmj.com).

If you have any questions on BMJ Open's open peer review process please email info.bmjopen@bmj.com

BMJ Open

Low-density lipoprotein cholesterol and all-cause mortality: findings from the China Health and Retirement Longitudinal Study

Journal:	*BMJ Open*
Manuscript ID	bmjopen-2020-036976
Article Type:	Original research
Date Submitted by the Author:	14-Jan-2020

Complete List of Authors:	Zhou, Liang; Liyang Center for Disease Control and Prevention<br>Wu, Ying; Southern Medical University, State Key Laboratory of Organ Failure Research, Department of Biostatistics, Guangdong Provincial Key Laboratory of Tropical Disease Research, School of Public Health<br>Yu, Shaobo; Soochow University Medical College<br>shen, yueping; Soochow University Medical College, Department of Epidemiology and Biostatistics<br>Ke, Chaofu; Soochow University Medical College, Department of Epidemiology and Biostatistics

Keywords:	Risk management < HEALTH SERVICES ADMINISTRATION & MANAGEMENT, PREVENTIVE MEDICINE, PUBLIC HEALTH, EPIDEMIOLOGY

SCHOLARONE™
Manuscripts

BMJ

I, the Submitting Author has the right to grant and does grant on behalf of all authors of the Work (as defined in the below author licence), an exclusive licence and/or a non-exclusive licence for contributions from authors who are: i) UK Crown employees; ii) where BMJ has agreed a CC-BY licence shall apply, and/or iii) in accordance with the terms applicable for US Federal Government officers or employees acting as part of their official duties; on a worldwide, perpetual, irrevocable, royalty-free basis to BMJ Publishing Group Ltd ("BMJ") its licensees and where the relevant Journal is co-owned by BMJ to the co-owners of the Journal, to publish the Work in this journal and any other BMJ products and to exploit all rights, as set out in our licence.

The Submitting Author accepts and understands that any supply made under these terms is made by BMJ to the Submitting Author unless you are acting as an employee on behalf of your employer or a postgraduate student of an affiliated institution which is paying any applicable article publishing charge ("APC") for Open Access articles. Where the Submitting Author wishes to make the Work available on an Open Access basis (and intends to pay the relevant APC), the terms of reuse of such Open Access shall be governed by a Creative Commons licence – details of these licences and which Creative Commons licence will apply to this Work are set out in our licence referred to above.

Other than as permitted in any relevant BMJ Author's Self Archiving Policies, I confirm this Work has not been accepted for publication elsewhere, is not being considered for publication elsewhere and does not duplicate material already published. I confirm all authors consent to publication of this Work and authorise the granting of this licence.

For peer review only

Low-density lipoprotein cholesterol and all-cause mortality: findings from the China Health and Retirement Longitudinal Study

Liang Zhou [1†], Ying Wu [2†], Shaobo Yu [3], Yueping Shen [4*] and Chaofu Ke [4*]

[1] Liyang Center for Disease Control and Prevention, 55 Nanhuan Road, Liyang 213371, P. R. China.

[2] State Key Laboratory of Organ Failure Research, Department of Biostatistics, Guangdong Provincial Key Laboratory of Tropical Disease Research, School of Public Health, Southern Medical University, Guangzhou 510515, China.

[3] Medical College of Soochow University, 199 Renai Road, Suzhou 215123, P. R. China.

[4] Department of Epidemiology and Biostatistics, School of Public Health, Medical College of Soochow University, 199 Renai Road, Suzhou 215123, P. R. China.

[†] Liang Zhou and Ying Wu made equal contributions to this work.

[*]Correspondence should be addressed to:

Chaofu Ke, Ph.D.

Tel.: +86-512-6588-0079

Fax: +86-512-6588-3323

E-mail: cfke@suda.edu.cn

Department of Epidemiology and Biostatistics, School of Public Health, Medical College of Soochow University, 199 Renai Road, Suzhou 215123, P. R. China.

or Yueping Shen, Ph.D.

Tel.: +86-512-6588-0079

Fax: +86-512-6588-3323

E-mail: shenyueping@suda.edu.cn

Department of Epidemiology and Biostatistics, School of Public Health, Medical College of Soochow University, 199 Renai Road, Suzhou 215123, P. R. China.

For peer review only

For peer review only - http://bmjopen.bmj.com/site/about/guidelines.xhtml

Abstract

Objectives

To investigate the relationship between LDL-C and all-cause mortality among middle-aged and elderly Chinese population.

Design

Prospective cohort study.

Setting

This study used data from the Chinese Health and Retirement Longitudinal Study (CHARLS).

Participants

Middle-aged and elderly participants with complete data were enrolled for a 4-year follow-up of total mortality and plasma levels of LDL-C, including 4983 male respondents and 5535 female respondents.

Results

During a 4-year follow-up, there were 305 and 219 deaths in men and women, respectively. Compared with the first quintile (Q1) of LDL-C, the adjusted hazard ratios (95% confidence intervals) were 0.832 (0.571~1.214) for Q2, 0.752 (0.509~1.110) for Q3, 0.555 (0.364~0.844) for Q4 and 0.643 (0.422~0.979) for Q5 in men. The results from restricted cubic spine (RCS) showed that when the 20th percentile of LDL-C levels was used as the reference, a lower LDL-C concentration was associated with a higher 4-year all-cause mortality risk. By contrast, both quintile analysis and RCS analysis showed a trend for increased mortality in women with low LDL-C concentrations, but the association was not statistically significant.

Conclusions

We found that a low level of LDL-C was associated with an increased risk of 4-year all-cause mortality in middle-aged and elderly Chinese men. The results suggest the potential harmful effect of a low level of LDL-C on total mortality.

Keywords Low-density lipoprotein cholesterol, all-cause mortality, CHARLS

For peer review only

For peer review only - http://bmjopen.bmj.com/site/about/guidelines.xhtml

Strengths and limitations of this study

- This study aimed to investigate the relationship between LDL-C and all-cause mortality among middle-aged and elderly Chinese population, based on high-quality data from a nationally representative longitudinal cohort.
- A low level of LDL-C was found to be associated with an increased risk of 4-year all-cause mortality in middle-aged and elderly Chinese men.
- Although the public's attention focuses on the benefit of lipid lowering, this study highlights the potential harmful effect of low LDL-C.
- The 4-year follow-up period prevented assessing the long-term association between LDL-C and all-cause mortality.
- The cause-specific mortality data were not available, preventing the analysis of the association between LDL-C and cause-specific mortality.

For peer review only

Introduction

For decades, the mainstream view holds that a high level of low-density lipoprotein cholesterol (LDL-C) is a primary cause of cardiovascular events and mortality[1]. However, many studies have found that LDL-C levels are inversely associated with all-cause mortality in diseased populations, such as patients with chronic hemodialysis[2], intracerebral hemorrhage[3] and heart failure[4]. Most importantly, in a systematic review of 19 cohort studies including 30 cohorts with 68 094 elderly people (≥60 years), an inverse association between LDL-C and all-cause mortality was seen in 16 cohorts representing 92% of all participants, and none of the other cohorts found the positive association between LDL-C and all-cause mortality[5]. Since LDL-C has been regarded as “bad cholesterol” for a long time and lipid-lowering drugs have been prescribed even at normal levels of serum cholesterol[6], the impact can be substantial. Therefore, the underlying relationship between LDL-C and all-cause mortality needs to be clarified in large prospective cohorts.

For peer review only - http://bmjopen.bmj.com/site/about/guidelines.xhtml

In this study, we aimed to investigate whether LDL-C levels are associated with all-cause mortality among the middle-aged and elderly Chinese men and women, based on the longitudinal data from the China Health and Retirement Longitudinal Study (CHARLS).

Methods

Study design

As a nationally representative longitudinal study, CHARLS is designed to collect a wide range of information on the economic standing, physical and psychological health, demographics and social networks of a middle-aged and elderly Chinese population (aged ≥45years)[7]. The national baseline survey (wave 1) was conducted between June 2011 and March 2012 and included 17,708 respondents. The second wave (wave 2) was carried out in 2013-2014, the third wave (wave 3) in 2014-2015 and the fourth wave (wave 4) in 2015-2016. The detailed design of CHARLS can be referred to a previous publication[7]. This study was approved by Biomedical Ethics Review Committee of Peking University, and all participants signed informed consents.

Study population

All participants recruited in the national baseline survey were included if they met the following criteria: 1) aged ≥45years, 2) measured plasma levels of LDL-C in wave 1, 3) successfully followed up in at least one of the subsequent three waves, and 4) without lipid-lowering interventions. Finally, 10518 participants, including 4983 men and 5535 women, were included for subsequent analysis (Figure 1).

Plasma LDL-C measurements and other covariates

Plasma samples were collected by medically-trained staff and then stored at –80°C until assayed at Capital Medical University (CMU) laboratory. LDL-C was measured by the enzymatic colormetric test, with an analytical range of 3–400 mg/L and between-assay coefficient of variation of 1.20%. During the testing of the CHARLS study samples, quality control (QC) samples were used daily. All test results from QC samples were within two standard deviations of mean QC control

For peer review only - http://bmjopen.bmj.com/site/about/guidelines.xhtml

concentrations. The other covariates collected included age, gender, smoking status, drinking status, body mass index (BMI), hypertension (defined by a history of hypertension, or systolic blood pressure (SBP) ≥ 140mmHg, or diastolic blood pressure (DBP) ≥ 90 mmHg), high blood sugar (HBS)/diabetes (defined by a history of HBS/diabetes, or fasting blood glucose ≥ 6.1mmol/L, or non-fasting blood glucose ≥ 7.8mmol/L), a history of cancer, cardiovascular disease, stroke, asthma, lung disease, liver disease, digestive disease, kidney disease, arthritis, memory problem and psychological problem.

All-cause mortality follow-up

Participants enrolled in wave 1 were followed up in subsequent three waves. In wave 2, both the interview status (dead or alive) and death time were recorded. In waves 3 and 4, only the interview status was recorded. For those who had the exact time on all-cause death, the survival time was defined as the interval between the interview time of wave 1 and the death time. If the exact death time was not available, the survival time was computed as the median of the interval between wave 1 and the specific wave with death information. For those who did not die during the follow-up period, the survival time was defined as the interval between two interview waves.

Patient and public involvement

Anonymised participant data were used in this study. Patients and the public were not involved in the design or conduct, or reporting, or dissemination plans of the study.

Statistical analysis

Data were presented as median (P_{25}~P_{75}) for continuous variables and frequency (percentage) for categorical variables. Baseline characteristics between or among groups were compared by the Wilcoxon rank sum test or Kruskal-Wallis rank sum test for continuous variables and by the chi-square test for categorical variables. The Cox proportional hazard ratio model was used to estimate the hazard ratios (HRs) and 95% confidence intervals (CIs) of LDL-C quintiles. In addition, the association between all-cause mortality and LDL-C on a continuous scale was further examined using restricted cubic splines (RCS) incorporated in Cox proportional hazards models.

For peer review only - http://bmjopen.bmj.com/site/about/guidelines.xhtml

All statistical analyses were performed by SAS statistical software (version 9.4, Cary, NC). All *P* values were 2-tailed, and the significance level was set at 0.05.

Results

Baseline characteristics of the study population

A total of 4983 men and 5535 women were eligible for the final analysis. The median of LDL-C levels in women was significantly higher than that in men ($P < 0.0001$, Supplementary Figure S1). Compared with women, men were older, had smaller BMI values and possessed greater smoking rate and drinking rate (all $P < 0.0001$). The prevalence rates of cancer, lung disease, arthritis and digestive disease in women were higher than those in men, but the prevalence rates of asthma and cardiovascular disease were lower in women (all $P < 0.0001$, Supplementary Table S1).

Characteristics of men and women according to the quintiles of LDL-C levels

After stratification by the quintiles of LDL-C levels, BMI, SBP and DBP in men were elevated with ascending quintiles as a whole (All $P<0.001$) (Supplementary Table S2). The prevalence of HBS/diabetes was highest in the bottom quintile of LDL-C and lowest in the fourth quintile, with prevalence rates of 34.94% and 27.02% respectively. There were no statistical differences among LDL-C quintiles for the other characteristics (e.g. age, smoking, drinking, stroke, cancer, cardiovascular disease, lung disease, liver disease, kidney disease, digestive disease, asthma, arthritis, psychological problem and memory problem) (All $P> 0.05$).

In women, LDL-C quintiles were positively associated with age, BMI, SBP and DBP (All $P <0.001$) (Supplementary Table S3). The prevalence rates of HBS/diabetes and liver disease in women were significantly different among different LDL-C quintiles (All $P<0.001$). For the remaining variables (e.g., smoking, drinking, stroke, cancer, cardiovascular disease, lung disease, kidney disease, digestive disease, asthma, arthritis, psychological problem and memory problem), no differences were observed (All $P > 0.05$).

Associations of LDL-C levels with all-cause mortality

For peer review only - http://bmjopen.bmj.com/site/about/guidelines.xhtml

In men, 305 out of 4983 participants died during a four-year follow-up. The mortality rates were declining with ascending quintiles (Table 1). Compared with the first quintile, the univariate HRs (95%CIs) were 0.738(0.537~1.014) for the second quintile; 0.638(0.457~0.890) for the third quintile; 0.521(0.366~0.742) for the fourth quintile and 0.511(0.358~0.730) for the fifth quintile. After adjustment for a series of potential confounders, the non-linear association between LDL-C and all-cause mortality was observed. As compared with the first quintile, the multivariate HRs (95%CIs) were as follows: second quintile, 0.834(0.572~1.216); third quintile, 0.752(0.510~1.110); fourth quintile, 0.555(0.365~0.845); fifth quintile, 0.643(0.422~0.980). In women, there were 219 deaths during a four-year follow-up. The mortality rate was highest in the first quintile. After adjustment for potential confounders, no quintile showed significant lower mortality rates compared with the first quintile (all $P > 0.05$) (Table 1).

The quintile analysis indicated that the relationship between LDL-C with all-cause mortality might be non-linear. Therefore, RCS was further performed to investigate the association between all-cause mortality and LDL-C on a continuous scale. The results from RCS showed that when the 20th percentile of LDL-C levels was used as the reference, lower LDL-C was associated with higher risk of 4-year all-cause mortality in men, and moderately higher LDL-C possessed lower total mortality risk, but the association was not statistically significant for very high LDL-C concentrations (Figure 2). For women, LDL-C was not significantly associated with 4-year all-cause mortality, but women at lower LDL-C concentrations were observed with a trend of a higher risk of 4-year total mortality (Figure 2).

Discussion

In this study, we investigated the relationship between LDL-C and 4-year all-cause mortality among the middle-aged and elderly Chinese population. In men, a low level of LDL-C was associated with increased mortality risk. In women, LDL-C was not significantly associated with 4-year all-cause mortality.

Low-density lipoprotein has been well established as an important cause of

For peer review only - http://bmjopen.bmj.com/site/about/guidelines.xhtml

cardiovascular disease (CVD) for decades[1]. Since CVD is the leading cause of mortality throughout the world, it is logically reasonable that increased LDL-C should contribute to increased CVD mortality and possibly all-cause mortality. Indeed, evidences from prospective epidemiologic studies showed a positive association between non-HDL-C concentration and ischaemic heart disease mortality[8]. However, non-HDL-C includes both LDL-C and very low-density lipoprotein cholesterol (VLDL-C). It is surprising that the direct association of LDL-C with CVD mortality was not consistently reported among studies. Abdullah *et al.* (2018) demonstrated that LDL-C was independently associated with CVD mortality in a low 10-year risk cohort with long-term follow-up[9]. By contrast, Tikhonof *et al.* (2005) reported that the elderly subjects (≥ 65 years) possessed the highest CVD mortality in the lowest LDL-C quartile[10]. Meanwhile, many other studies also found no association between LDL-C and CVD mortality[11-13]. When the results about the association of LDL-C with CVD mortality were inconsistent, it is more surprising to find that few studies have reported the positive association between LDL-C and all-cause mortality. In the study by Abdullah *et al.* (2018), there were already no associations or minimal positive associations between high LDL-C categories and all-cause mortality even in univariable Cox analyses[9]. Most remarkably, results of multivariable Cox analyses in this study were not provided for all-cause mortality, which could exert a substantial impact on the final association[14]. Actually, a large number of studies reported no association or even an inverse association between LDL-C and all-cause mortality, which has been summarized in a systematic review by Ravnskov *et al.* (2016)[5]. Therefore, although the mainstream view has been advocating the benefit of lowering high LDL-C, the harmful effect of low LDL-C may be largely neglected.

It should be noted that the confounding effect of statin treatment should be minimized, as this study excluded those who used lipid-lowering interventions. There was also no association between baseline LDL-C and the presence of cancer, stroke and CVD neither among men nor among women (Supplementary Tables S2 and S3). Moreover, when participants who had died during the first observation year were

For peer review only - http://bmjopen.bmj.com/site/about/guidelines.xhtml

excluded, this relationship was not changed. This could relieve the concern that serious diseases may lower cholesterol soon before death occurs. We speculate that the difference between men and women was due to the much lower LDL-C levels in males than in females. This could result in the small sample size of female participants with low LDL-C concentrations, leading to insufficient power for the association in women. Indeed, there was observed a high risk trend for women at low LDL-C concentrations, although the association was not statistically significant.

Several explanations for the unfavorable effects of low LDL-C levels may be proposed. LDL-C has been suggested to play an important role in host defense against both bacterial and viral pathogens[15]. Indeed, many animal and laboratory experiments have shown that LDL could bind to and inactivate a broad range of microorganisms and their toxic products[16-18]. This hypothesis may be further supported by the recent finding that LDL-C was associated with reduced infectious mortality based on the data from 37,250 patients in the international Monitoring Dialysis Outcomes (MONDO) database[2]. In addition, it has been proposed that LDL-C may have the potential to protect against cancer as many cancer types are caused by viruses[19]. Ravnskov *et al.* (2012) reviewed nine cohort studies including more than 140,000 individuals followed for 10–30 years and found that low cholesterol was associated with cancer[20]. Moreover, cholesterol-lowering experiments on rodents have led to cancer as well[21]. In agreement with these findings, individuals with familial hypercholesterolaemia have been found to possess significantly lower cancer mortality[22]. Therefore, lower LDL-C may contribute to a higher risk of death from infection and cancer, which in turn results in increased all-cause mortality.

This study demonstrated that middle-aged and elderly Chinese men with low LDL-C had an increased risk of all-cause mortality, which calls for special attention to be paid to the possible harmful effect of a low level of LDL-C. However, some limitations should be noted. First, the follow-up period was limited to 4 years. For a longer follow-up time, the associations between LDL-C and all-cause mortality in women might be displayed. In addition, cause-specific mortality data were not

For peer review only - http://bmjopen.bmj.com/site/about/guidelines.xhtml

available for the time being, preventing the analysis of the association between LDL-C and cause-specific mortality.

In China, 4-year total mortality is associated with a low level of plasma LDL-C in middle-aged and elderly men. The findings in this study may suggest the potential harmful effect of a low level of LDL-C. More prospective and well-designed studies are needed to validate the relationship between LDL-C and mortality.

Acknowledgements

This analysis uses data or information from the Harmonized CHARLS dataset and Codebook, Version C as of April 2018 developed by the Gateway to Global Aging Data. The development of the Harmonized CHARLS was funded by the National Institute on Ageing (R01 AG030153, RC2 AG036619, R03 AG043052). For more information, please refer to www.g2aging.org.

Funding

This work was supported by National Natural Science Foundation of China (81703316 to C.K., 81703322 to Y.W.) and Natural Science Foundation of Jiangsu Province (BK20170350).

Competing interests

None declared.

Patient consent for publication

Obtained

Contributors

CK and YS conceived and designed the research; LZ and CK wrote the manuscript; and YW and SY performed the data analysis. All authors contributed to the interpretations of the findings. All authors reviewed the manuscript.

For peer review only - http://bmjopen.bmj.com/site/about/guidelines.xhtml

Ethics approval

CHARLS was approved by Biomedical Ethics Review Committee of Peking University, and all participants signed informed consents.

Data sharing statement

The data used and analysed in this study are publicly available from the China Health and Retirement Longitudinal Study (http://charls.pku.edu.cn/zh-CN).

Figure legends

Figure 1 Flowchart on the selection of eligible participants.

Figure 2 Results from restricted cubic splines for the association between LDL-C and 4-year all-cause mortality in men and women, respectively.

References

1. Ference BA, Ginsberg HN, Graham I, et al. Low-density lipoproteins cause atherosclerotic cardiovascular disease. 1. Evidence from genetic, epidemiologic, and clinical studies. A consensus statement from the European Atherosclerosis Society Consensus Panel. Eur Heart J 2017;**38**(32):2459-72 |.
2. Kaysen GA, Ye X, Raimann JG, et al. Lipid levels are inversely associated with infectious and all-cause mortality: international MONDO study results. Journal of lipid research 2018;**59**(8):1519-28|.
3. Chang JJ, Katsanos AH, Khorchid Y, et al. Higher low-density lipoprotein cholesterol levels are associated with decreased mortality in patients with intracerebral hemorrhage. Atherosclerosis 2018;**269**:14-20|.
4. Charach G, George J, Roth A, et al. Baseline low-density lipoprotein cholesterol levels and outcome in patients with heart failure. The American journal of cardiology 2010;**105**(1):100-4|.
5. Ravnskov U, Diamond DM, Hama R, et al. Lack of an association or an inverse association between low-density-lipoprotein cholesterol and mortality in the elderly: a systematic review. Bmj Open 2016;**6**(6): e010401.
6. Heart Protection Study Collaborative G. MRC/BHF Heart Protection Study of cholesterol lowering with simvastatin in 20,536 high-risk individuals: a randomised placebo-controlled trial. Lancet 2002;**360**(9326):7-22|.
7. Zhao Y, Hu Y, Smith JP, et al. Cohort profile: the China Health and Retirement Longitudinal Study (CHARLS). Int J Epidemiol 2014;**43**(1):61-8|.
8. Prospective Studies C, Lewington S, Whitlock G, et al. Blood cholesterol and vascular mortality by age, sex, and blood pressure: a meta-analysis of individual data from 61 prospective studies with 55,000 vascular deaths. Lancet 2007;**370**(9602):1829-39|.

For peer review only - http://bmjopen.bmj.com/site/about/guidelines.xhtml

9. Abdullah SM, Defina LF, Leonard D, et al. Long-Term Association of Low-Density Lipoprotein Cholesterol With Cardiovascular Mortality in Individuals at Low 10-Year Risk of Atherosclerotic Cardiovascular Disease. Circulation 2018;**138**(21):2315-25|.
10. Tikhonoff V, Casiglia E, Mazza A, et al. Low-density lipoprotein cholesterol and mortality in older people. Journal of the American Geriatrics Society 2005;**53**(12):2159-64|.
11. Raiha I, Marniemi J, Puukka P, et al. Effect of serum lipids, lipoproteins, and apolipoproteins on vascular and nonvascular mortality in the elderly. Arteriosclerosis, thrombosis, and vascular biology 1997;**17**(7):1224-32.
12. Upmeier E, Lavonius S, Lehtonen A, et al. Serum lipids and their association with mortality in the elderly: a prospective cohort study. Aging Clin Exp Res 2009;**21**(6):424-30|.
13. Blekkenhorst LC, Prince RL, Hodgson JM, et al. Dietary saturated fat intake and atherosclerotic vascular disease mortality in elderly women: a prospective cohort study. The American journal of clinical nutrition 2015;**101**(6):1263-8|.
14. Ke CF, Shen YP. Letter by Ke and Shen Regarding Article, "Long-Term Association of Low-Density Lipoprotein Cholesterol With Cardiovascular Mortality in Individuals at Low 10-Year Risk of Atherosclerotic Cardiovascular Disease: Results From the Cooper Center Longitudinal Study". Circulation 2019;**139**(18):2190-91|.
15. Feingold KR, Grunfeld C. Lipids: a key player in the battle between the host and microorganisms. Journal of lipid research 2012;**53**(12):2487-89|.
16. Han RL. Plasma lipoproteins are important components of the immune system. Microbiol Immunol 2010;**54**(4):246-53|.
17. Khovidhunkit W, Kim MS, Memon RA, et al. Effects of infection and inflammation on lipid and lipoprotein metabolism: mechanisms and consequences to the host. Journal of lipid research 2004;**45**(7):1169-96|.
18. Ravnskov U, McCully KS. Review and Hypothesis: Vulnerable plaque formation from obstruction of Vasa vasorum by homocysteinylated and oxidized lipoprotein aggregates complexed with microbial remnants and LDL autoantibodies. Annals of clinical and laboratory science 2009;**39**(1):3-16.
19. Read SA, Douglas MW. Virus induced inflammation and cancer development. Cancer letters 2014;**345**(2):174-81|.
20. Ravnskov U, McCully KS, Rosch PJ. The statin-low cholesterol-cancer conundrum. QJM : monthly journal of the Association of Physicians 2012;**105**(4):383-8.
21. Newman TB, Hulley SB. Carcinogenicity of lipid-lowering drugs. Jama 1996;**275**(1):55-60.
22. Neil HA, Hawkins MM, Durrington PN, et al. Non-coronary heart disease mortality and risk of fatal cancer in patients with treated heterozygous familial hypercholesterolaemia: a prospective registry study. Atherosclerosis 2005;**179**(2):293-7.

For peer review only

Table 1 Associations between LDL-C and all-cause mortality

	Total	Deaths (%)	Unadjusted		Adjusted*	
			HR (95%CI)	*P* value	HR (95%CI)	*P* value
Men						
Q1	991	88(8.88)	1	-	1	-
Q2	1008	67(6.65)	0.733(0.533~1.007)	0.0554	0.832(0.571~1.214)	0.3408
Q3	992	57(5.75)	0.638(0.457~0.891)	0.0083	0.752(0.509~1.110)	0.1512
Q4	1004	47(4.68)	0.519(0.364~0.739)	0.0003	0.555(0.364~0.844)	0.0060
Q5	988	46(4.66)	0.512(0.358~0.731)	0.0002	0.643(0.422~0.979)	0.0397
Women						
Q1	1117	52(4.66)	1	-	1	-
Q2	1102	49(4.45)	0.963(0.652~1.423)	0.8505	1.172(0.732~1.876)	0.5090
Q3	1097	29(2.64)	0.566(0.360~0.892)	0.0141	0.612(0.353~1.061)	0.0800
Q4	1112	41(3.69)	0.793(0.527~1.194)	0.2671	0.836(0.511~1.369)	0.4774
Q5	1107	48(4.34)	0.928(0.627~1.373)	0.7077	0.859(0.533~1.384)	0.5324

*Adjusted for age, smoking, drinking, BMI, hypertension, HBS/diabetes, history of stroke, cancer, cardiovascular disease, lung disease, liver disease, kidney disease, digestive disease, asthma, arthritis, psychological problem and memory problem.

For peer review only

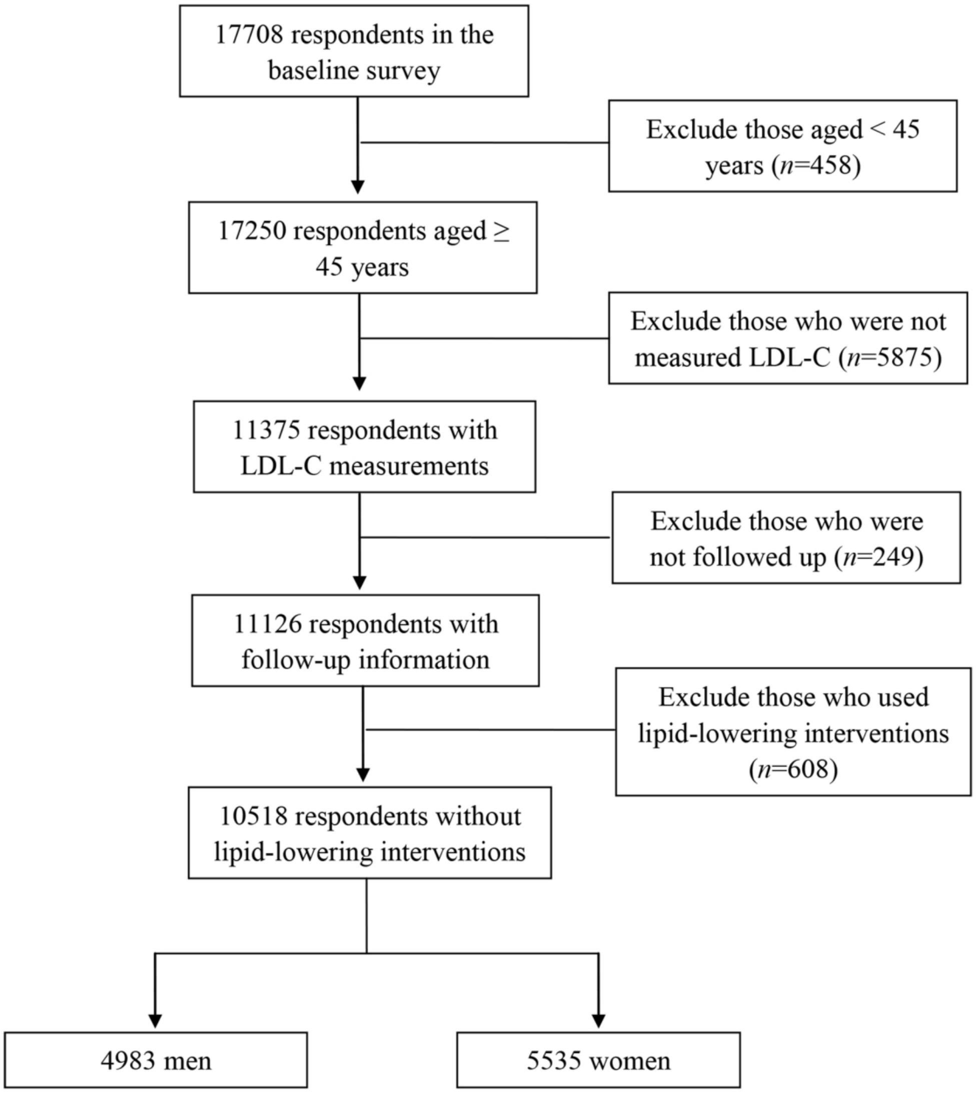

Figure 1 Flowchart on the selection of eligible participants.

151x167mm (300 x 300 DPI)

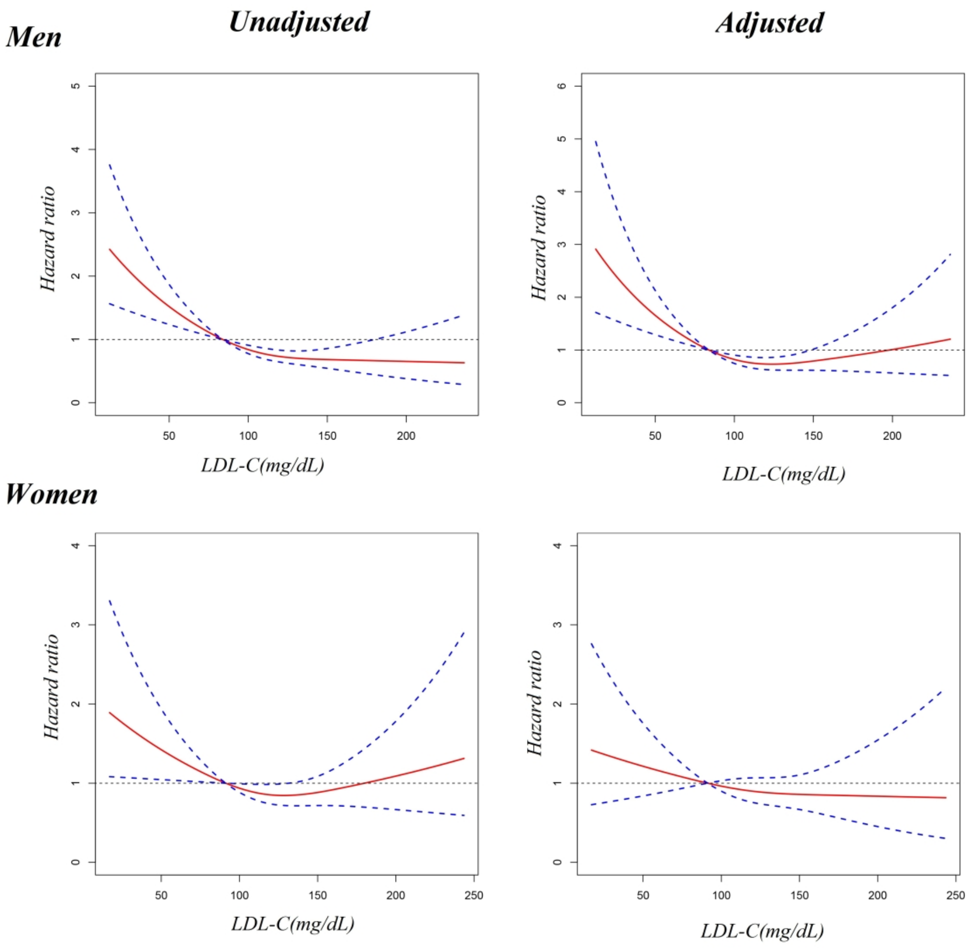

Figure 2 Results from restricted cubic splines for the association between LDL-C and 4-year all-cause mortality in men and women, respectively.

377x370mm (300 x 300 DPI)

Supplementary materials

Low-density lipoprotein cholesterol and all-cause mortality: findings from the China Health and Retirement Longitudinal Study

Liang Zhou [1], Ying Wu [2], Shaobo Yu [3], Yueping Shen [4] and Chaofu Ke [4]

[1] Liyang Center for Disease Control and Prevention, 55 Nanhuan Road, Liyang 213371, P. R. China.

[2] State Key Laboratory of Organ Failure Research, Department of Biostatistics, Guangdong Provincial Key Laboratory of Tropical Disease Research, School of Public Health, Southern Medical University, Guangzhou 510515, China.

[3] Medical College of Soochow University, Suzhou 215123, P. R. China.

[4] Department of Epidemiology and Biostatistics, School of Public Health, Medical College of Soochow University, 199 Renai Road, Suzhou 215123, P. R. China.

For peer review only

Supplementary Table S1 Characteristics of the study population

Characteristiscs	Men (*n*=4983)	Women (*n*=5535)	*P* value
Age-yr	59 (53~66)	57 (51~65)	<0.0001
BMI-kg/m^2	22.40 (20.35~24.83)	23.51 (21.18~26.14)	<0.0001
SBP-mmHg	127.67 (115.67~141.67)	127.00 (114.00~143.33)	0.3762
DBP-mmHg	75.33 (67.67~83.67)	74.33 (67.00~82.67)	0.0017
Lifestyle-no. (%)			
Smoking ever	3738 (75.24)	420 (7.62)	<0.0001
Drinking ever	3297 (66.43)	826 (15.00)	<0.0001
Disease history-no. (%)			
Hypertension	1942 (44.15)	2290 (46.21)	0.0457
HBS/Diabetes	1461 (30.17)	1565 (29.20)	0.2840
Cancer	40 (0.81)	67 (1.22)	0.0373
Stroke	133 (2.69)	136 (2.47)	0.4948
Cardiovascular disease	499 (10.10)	738 (13.47)	<0.0001
Lung disease	620 (12.54)	508 (9.26)	<0.0001
Arthritis	1548 (31.23)	2243 (40.80)	<0.0001
Liver disease	197 (4.00)	188 (3.44)	0.1321
Kidney disease	309 (6.27)	315 (5.75)	0.2691
Digestive disease	1030 (20.79)	1394 (25.38)	<0.0001
Asthma	283 (5.72)	216 (3.93)	<0.0001
Psychological problem	60 (1.21)	94 (1.72)	0.0337
Memory problem	97 (1.96)	86 (1.57)	0.1235

Note: BMI, body mass index; SBP, systolic blood pressure; DBP, diastolic blood pressure; HBS, high blood sugar.

For peer review only

Supplementary Table S2 Baseline characteristics of participants by quintiles of LDL-C in men

Characteristics	Quintile 1 (*n*=991) (≤83.89mg/dL)	Quintile 2 (*n*=1008) (83.89~101.68)	Quintile 3 (*n*=992) (101.68~117.14)	Quintile 4 (*n*=1004) (117.14~136.86)	Quintile 5 (*n*=988) (>136.86)	*P* value
Age-yr	59 (52~66)	59 (53~67)	59 (52~66)	59 (53~67)	59 (53~65)	0.5405
BMI-kg/m^2	21.57 (19.78~24.21)	22.18 (20.31~24.39)	22.36 (20.44~24.61)	22.64 (20.65~25.07)	23.27 (20.95~25.68)	<0.0001
SBP-mmHg	126.00 (114.00~140.00)	126.00 (115.33~139.67)	127.00 (114.67~142.00)	128.00 (115.67~142.50)	130.67 (118.00~144.00)	0.0002
DBP-mmHg	75.00 (66.67~83.33)	74.33 (66.67~82.00)	74.67 (67.33~83.33)	75.33 (67.67~83.67)	76.67 (69.33~85.33)	0.0002
Lifestyle-no. (%)						
Smoking ever	755 (76.57)	754 (75.02)	751 (75.94)	759 (75.75)	719 (72.92)	0.3788
Drinking ever	654 (66.40)	663 (66.10)	664 (67.14)	664 (66.40)	652 (66.13)	0.9890
Disease history-no. (%)						
Hypertension	373 (43.52)	354 (40.05)	394 (43.83)	402 (45.07)	419 (48.33)	0.0131
HBS/Diabetes	337 (34.81)	277 (28.35)	267 (27.93)	266 (27.06)	314 (32.78)	0.0003
Cancer	13 (1.32)	7 (0.70)	5 (0.51)	6 (0.60)	9 (0.92)	0.2728
Stroke	26 (2.64)	33 (3.31)	21 (2.13)	25 (2.50)	28 (2.85)	0.5818
Cardiovascular disease	86 (8.78)	98 (9.81)	103 (10.47)	113 (11.31)	99 (10.10)	0.4453
Lung disease	131 (13.37)	135 (13.46)	106 (10.77)	120 (12.00)	128 (13.09)	0.3170
Arthritis	300 (30.43)	332 (33.10)	280 (28.34)	312 (31.23)	324 (33.03)	0.1233
Liver disease	51 (5.23)	42 (4.19)	35 (3.57)	38 (3.81)	31 (3.18)	0.1855
Kidney disease	56 (5.73)	73 (7.29)	59 (6.02)	57 (5.71)	64 (6.56)	0.5518
Digestive disease	211 (21.46)	213 (21.24)	225 (22.82)	203 (20.28)	178 (18.14)	0.1264
Asthma	66 (6.76)	49 (4.89)	43 (4.36)	59 (5.91)	66 (6.72)	0.0754
Psychological problem	14 (1.43)	10 (1.00)	14 (1.42)	14 (1.40)	8 (0.82)	0.6092
Memory problem	25 (2.55)	13 (1.30)	21 (2.13)	19 (1.90)	19 (1.94)	0.3810

Note: BMI, body mass index; SBP, systolic blood pressure; DBP, diastolic blood pressure; HBS, high blood sugar.

For peer review only

Supplementary Table S3 Baseline characteristics of participants by quintiles of LDL-C in women

Characteristics	Quintile 1 (*n*=1117) (≤91.24mg/dL)	Quintile 2 (*n*=1102) (91.24~109.41)	Quintile 3 (*n*=1097) (109.41~126.03)	Quintile 4 (*n*=1112) (126.03~147.49)	Quintile 5 (*n*=1107) (>147.49)	*P* value
Age-yr	56 (49~64)	56 (49~64)	57 (50~63)	58 (52~65)	59 (53~66)	<0.0001
BMI-kg/m^2	23.15 (20.90~25.76)	23.30 (21.01~25.82)	23.39 (21.10~26.02)	23.63 (21.32~26.26)	24.12 (21.57~26.83)	<0.0001
SBP-mmHg	124.67 (112.00~141.00)	125.67 (113.00~142.33)	127.00 (114.67~143.00)	127.50 (114.83~142.33)	130.00 (117.00~146.00)	<0.0001
DBP-mmHg	73.33 (65.67~81.67)	74.33 (66.67~82.67)	74.00 (67.33~82.67)	74.67 (67.67~82.50)	75.33 (67.67~83.33)	0.0102
Lifestyle-no. (%)						
Smoking ever	71 (6.39)	93 (8.49)	78 (7.15)	79 (7.13)	99 (8.97)	0.1289
Drinking ever	173 (15.61)	160 (14.61)	169 (15.50)	165 (14.89)	159 (14.40)	0.9108
Disease history-no. (%)						
Hypertension	425 (42.71)	452 (45.80)	439 (44.98)	459 (46.22)	515 (51.24)	0.0033
HBS/Diabetes	310 (28.78)	290 (27.46)	280 (26.19)	312 (28.94)	373 (34.53)	0.0003
Cancer	14 (1.27)	15 (1.38)	18 (1.66)	14 (1.27)	6 (0.54)	0.1876
Stroke	31 (2.80)	16 (1.47)	27 (2.47)	31 (2.81)	31 (2.81)	0.1910
Cardiovascular disease	144 (13.08)	135 (12.41)	140 (12.89)	166 (15.08)	153 (13.87)	0.3924
Lung disease	110 (9.94)	112 (10.29)	103 (9.45)	91 (8.26)	92 (8.36)	0.3540
Arthritis	454 (41.05)	450 (41.21)	421 (38.55)	454 (41.01)	464 (42.14)	0.5201
Liver disease	53 (4.80)	40 (3.69)	25 (2.31)	43 (3.91)	27 (2.46)	0.0060
Kidney disease	74 (6.71)	62 (5.69)	57 (5.24)	66 (6.01)	56 (5.09)	0.4894
Digestive disease	279 (25.20)	267 (24.45)	269 (24.70)	300 (27.20)	279 (25.34)	0.6078
Asthma	43 (3.89)	43 (3.94)	44 (4.03)	39 (3.54)	47 (4.26)	0.9374
Psychological problem	16 (1.45)	19 (1.74)	23 (2.12)	17 (1.54)	19 (1.73)	0.7936
Memory problem	18 (1.63)	19 (1.74)	11 (1.01)	15 (1.36)	23 (2.09)	0.3240

Note: BMI, body mass index; SBP, systolic blood pressure; DBP, diastolic blood pressure; HBS, high blood sugar.

For peer review only

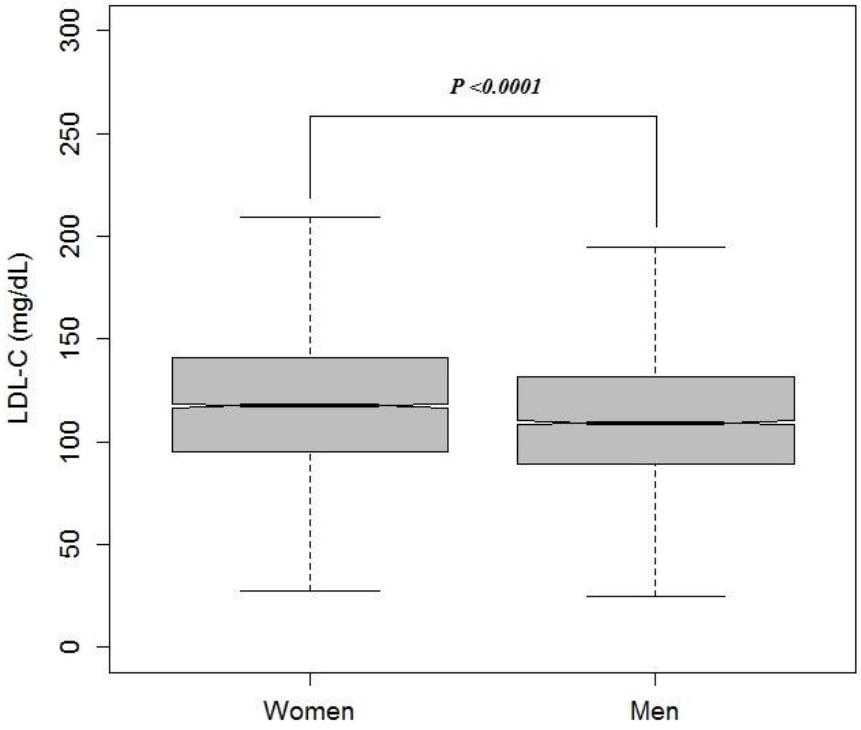

Supplementary Figure S1 The box-plot of plasma LDL-C levels in middle-aged and elderly Chinese men and women.

STROBE 2007 (v4) Statement—Checklist of items that should be included in reports of *cohort studies*

Section/Topic	Item #	Recommendation	Reported on page #
**Title and abstract**	1	(*a*) Indicate the study's design with a commonly used term in the title or the abstract	2
		(*b*) Provide in the abstract an informative and balanced summary of what was done and what was found	2
**Introduction**			
Background/rationale	2	Explain the scientific background and rationale for the investigation being reported	3
Objectives	3	State specific objectives, including any prespecified hypotheses	4
**Methods**			
Study design	4	Present key elements of study design early in the paper	4
Setting	5	Describe the setting, locations, and relevant dates, including periods of recruitment, exposure, follow-up, and data collection	4,5
Participants	6	(*a*) Give the eligibility criteria, and the sources and methods of selection of participants. Describe methods of follow-up	4,5
		(*b*) For matched studies, give matching criteria and number of exposed and unexposed	Not applicable
Variables	7	Clearly define all outcomes, exposures, predictors, potential confounders, and effect modifiers. Give diagnostic criteria, if applicable	4,5
Data sources/ measurement	8*	For each variable of interest, give sources of data and details of methods of assessment (measurement). Describe comparability of assessment methods if there is more than one group	*4,5*
Bias	9	Describe any efforts to address potential sources of bias	4,7,8,9
Study size	10	Explain how the study size was arrived at	4
Quantitative variables	11	Explain how quantitative variables were handled in the analyses. If applicable, describe which groupings were chosen and why	5
Statistical methods	12	(*a*) Describe all statistical methods, including those used to control for confounding	5
		(*b*) Describe any methods used to examine subgroups and interactions	5,6,7
		(*c*) Explain how missing data were addressed	4,5
		(*d*) If applicable, explain how loss to follow-up was addressed	5
		(*e*) Describe any sensitivity analyses	5
**Results**			

For peer review only

Participants	13*	(a) Report numbers of individuals at each stage of study—eg numbers potentially eligible, examined for eligibility, confirmed eligible, included in the study, completing follow-up, and analysed	4,5
		(b) Give reasons for non-participation at each stage	4,5
		(c) Consider use of a flow diagram	4
Descriptive data	14*	(a) Give characteristics of study participants (eg demographic, clinical, social) and information on exposures and potential confounders	6
		(b) Indicate number of participants with missing data for each variable of interest	4
		(c) Summarise follow-up time (eg, average and total amount)	5
Outcome data	15*	Report numbers of outcome events or summary measures over time	7
Main results	16	(*a*) Give unadjusted estimates and, if applicable, confounder-adjusted estimates and their precision (eg, 95% confidence interval). Make clear which confounders were adjusted for and why they were included	7
		(*b*) Report category boundaries when continuous variables were categorized	5,7
		(*c*) If relevant, consider translating estimates of relative risk into absolute risk for a meaningful time period	Not applicable
Other analyses	17	Report other analyses done—eg analyses of subgroups and interactions, and sensitivity analyses	6,7,9
**Discussion**			
Key results	18	Summarise key results with reference to study objectives	7
**Limitations**			
Interpretation	20	Give a cautious overall interpretation of results considering objectives, limitations, multiplicity of analyses, results from similar studies, and other relevant evidence	8,9,10
Generalisability	21	Discuss the generalisability (external validity) of the study results	8,9,10
**Other information**			
Funding	22	Give the source of funding and the role of the funders for the present study and, if applicable, for the original study on which the present article is based	10

*Give information separately for cases and controls in case-control studies and, if applicable, for exposed and unexposed groups in cohort and cross-sectional studies.

Note: An Explanation and Elaboration article discusses each checklist item and gives methodological background and published examples of transparent reporting. The STROBE checklist is best used in conjunction with this article (freely available on the Web sites of PLoS Medicine at http://www.plosmedicine.org/, Annals of Internal Medicine at http://www.annals.org/, and Epidemiology at http://www.epidem.com/). Information on the STROBE Initiative is available at www.strobe-statement.org.

For peer review only

BMJ Open

Low-density lipoprotein cholesterol and all-cause mortality: findings from the China Health and Retirement Longitudinal Study

Journal:	*BMJ Open*
Manuscript ID	bmjopen-2020-036976.R1
Article Type:	Original research
Date Submitted by the Author:	26-Apr-2020
Complete List of Authors:	Zhou, Liang; Liyang Center for Disease Control and Prevention<br>Wu, Ying; Southern Medical University, State Key Laboratory of Organ Failure Research, Department of Biostatistics, Guangdong Provincial Key Laboratory of Tropical Disease Research, School of Public Health<br>Yu, Shaobo; Soochow University Medical College<br>shen, yueping; Soochow University Medical College, Department of Epidemiology and Biostatistics<br>Ke, Chaofu; Soochow University Medical College, Department of Epidemiology and Biostatistics
**Primary Subject Heading**:	Epidemiology
Secondary Subject Heading:	Epidemiology, Public health
Keywords:	Risk management < HEALTH SERVICES ADMINISTRATION & MANAGEMENT, PREVENTIVE MEDICINE, PUBLIC HEALTH, EPIDEMIOLOGY

SCHOLARONE™
Manuscripts

I, the Submitting Author has the right to grant and does grant on behalf of all authors of the Work (as defined in the below author licence), an exclusive licence and/or a non-exclusive licence for contributions from authors who are: i) UK Crown employees; ii) where BMJ has agreed a CC-BY licence shall apply, and/or iii) in accordance with the terms applicable for US Federal Government officers or employees acting as part of their official duties; on a worldwide, perpetual, irrevocable, royalty-free basis to BMJ Publishing Group Ltd ("BMJ") its licensees and where the relevant Journal is co-owned by BMJ to the co-owners of the Journal, to publish the Work in this journal and any other BMJ products and to exploit all rights, as set out in our licence.

The Submitting Author accepts and understands that any supply made under these terms is made by BMJ to the Submitting Author unless you are acting as an employee on behalf of your employer or a postgraduate student of an affiliated institution which is paying any applicable article publishing charge ("APC") for Open Access articles. Where the Submitting Author wishes to make the Work available on an Open Access basis (and intends to pay the relevant APC), the terms of reuse of such Open Access shall be governed by a Creative Commons licence – details of these licences and which Creative Commons licence will apply to this Work are set out in our licence referred to above.

Other than as permitted in any relevant BMJ Author's Self Archiving Policies, I confirm this Work has not been accepted for publication elsewhere, is not being considered for publication elsewhere and does not duplicate material already published. I confirm all authors consent to publication of this Work and authorise the granting of this licence.

For peer review only

Low-density lipoprotein cholesterol and all-cause mortality: findings from the China Health and Retirement Longitudinal Study

Liang Zhou [1†], Ying Wu [2†], Shaobo Yu [3], Yueping Shen [4*] and Chaofu Ke [4*]

[1] Liyang Center for Disease Control and Prevention, 55 Nanhuan Road, Liyang 213371, P. R. China.

[2] State Key Laboratory of Organ Failure Research, Department of Biostatistics, Guangdong Provincial Key Laboratory of Tropical Disease Research, School of Public Health, Southern Medical University, Guangzhou 510515, China.

[3] Medical College of Soochow University, 199 Renai Road, Suzhou 215123, P. R. China.

[4] Department of Epidemiology and Biostatistics, School of Public Health, Medical College of Soochow University, 199 Renai Road, Suzhou 215123, P. R. China.

[†] Liang Zhou and Ying Wu made equal contributions to this work.

[*]Correspondence should be addressed to:

Chaofu Ke, Ph.D.

Tel.: +86-512-6588-0079

Fax: +86-512-6588-3323

E-mail: cfke@suda.edu.cn

Department of Epidemiology and Biostatistics, School of Public Health, Medical College of Soochow University, 199 Renai Road, Suzhou 215123, P. R. China.

or Yueping Shen, Ph.D.

Tel.: +86-512-6588-0079

Fax: +86-512-6588-3323

E-mail: shenyueping@suda.edu.cn

Department of Epidemiology and Biostatistics, School of Public Health, Medical College of Soochow University, 199 Renai Road, Suzhou 215123, P. R. China.

For peer review only

For peer review only - http://bmjopen.bmj.com/site/about/guidelines.xhtml

Abstract

Objectives

To investigate the relationship between low-density lipoprotein cholesterol (LDL-C) and all-cause mortality among middle-aged and elderly Chinese population.

Design

Prospective cohort study.

Setting

This study used data from the Chinese Health and Retirement Longitudinal Study (CHARLS).

Participants

Middle-aged and elderly participants with complete data were enrolled for a 4-year follow-up of total mortality and plasma levels of LDL-C, including 4981 male respondents and 5529 female respondents.

Results

During a 4-year follow-up, there were 305 and 219 deaths in men and women, respectively. Compared with the first quintile (Q1) of LDL-C, the adjusted hazard ratios (95% confidence intervals) were 0.866(0.567~1.325) for Q2, 0.782(0.507~1.206) for Q3, 0.577(0.363~0.916) for Q4 and 0.788(0.497~1.248) for Q5 in men. The results from restricted cubic spine (RCS) showed that when the 20th percentile of LDL-C levels was used as the reference, a lower LDL-C concentration was associated with a higher 4-year all-cause mortality risk. By contrast, both quintile analysis and RCS analysis did not show a statistically significant association in women.

Conclusions

We found that a very low plasma level of LDL-C was associated with an increased risk of 4-year all-cause mortality in middle-aged and elderly Chinese men. The results suggest the potential harmful effect of a quite low level of LDL-C on total mortality.

Keywords Low-density lipoprotein cholesterol, all-cause mortality, CHARLS

For peer review only - http://bmjopen.bmj.com/site/about/guidelines.xhtml

Strengths and limitations of this study

- This study used high-quality data from a nationally representative longitudinal cohort to investigate the relationship between LDL-C and all-cause mortality among middle-aged and elderly Chinese population.
- The use of restricted cubic spline provided a more comprehensive spectrum of the non-linear relation between LDL-C and all-cause mortality.
- Although the public's attention focuses on the benefit of lipid lowering, this study highlights the potential harmful effect of very low LDL-C.
- The 4-year follow-up period prevented the assessment of a long-term association between LDL-C and all-cause mortality.
- The unavailability of cause-specific mortality data prevented the analysis of the association between LDL-C and cause-specific mortality.

For peer review only

Introduction

For decades, the mainstream view holds that a high level of low-density lipoprotein cholesterol (LDL-C) is a primary cause of cardiovascular events and mortality[1]. However, many studies have found that LDL-C levels are inversely associated with all-cause mortality in diseased populations, such as patients with chronic hemodialysis[2], intracerebral hemorrhage[3] and heart failure[4]. Most importantly, in a systematic review of 19 cohort studies including 30 cohorts with 68 094 elderly people (≥60 years), an inverse association between LDL-C and all-cause mortality was seen in 16 cohorts representing 92% of all participants, and none of the other cohorts found the positive association between LDL-C and all-cause mortality[5]. Since LDL-C has been regarded as “bad cholesterol” for a long time and lipid-lowering drugs have been prescribed even at normal levels of serum cholesterol[6], the impact can be substantial. Therefore, the underlying relationship between LDL-C and all-cause mortality needs to be clarified in large prospective cohorts.

For peer review only - http://bmjopen.bmj.com/site/about/guidelines.xhtml

In this study, we aimed to investigate whether LDL-C levels are associated with all-cause mortality among middle-aged and elderly Chinese men and women, based on the longitudinal data from the China Health and Retirement Longitudinal Study (CHARLS).

Methods

Study design

As a nationally representative longitudinal study, CHARLS is designed to collect a wide range of information on the economic standing, physical and psychological health, demographics and social networks of a middle-aged and elderly Chinese population (aged ≥45years)[7]. The national baseline survey (wave 1) was conducted between June 2011 and March 2012 and included 17,708 respondents. The second wave (wave 2) was carried out in 2013-2014, the third wave (wave 3) in 2014-2015 and the fourth wave (wave 4) in 2015-2016. The detailed design of CHARLS can be referred to a previous publication[7]. This study was approved by Biomedical Ethics Review Committee of Peking University, and all participants signed informed consents.

Study population

All participants recruited in the national baseline survey were included if they met the following criteria: 1) aged ≥45years, 2) measured plasma levels of LDL-C in wave 1, 3) successfully followed up in at least one of the subsequent three waves, and 4) without lipid-lowering interventions. Finally, 10510 participants, including 4981 men and 5529 women, were included for subsequent analysis (Figure 1).

Plasma LDL-C measurements and other covariates

Plasma samples were collected by medically-trained staff and then stored at –80°C until assayed at Capital Medical University (CMU) laboratory. LDL-C was measured by the enzymatic colormetric test, with an analytical range of 3–400 mg/L and between-assay coefficient of variation of 1.20%. During the testing of the CHARLS study samples, quality control (QC) samples were used daily. All test results from QC samples were within two standard deviations of mean QC control

For peer review only

concentrations. The other covariates collected included age, gender, smoking status, drinking status, body mass index (BMI), educational level, household income, living alone status, rural residence, activity of daily living (ADL) disability, high-density lipoprotein cholesterol (HDL-C), triglyceride, hemoglobin, hypertension (defined by a history of hypertension, or systolic blood pressure (SBP) ≥ 140mmHg, or diastolic blood pressure (DBP) ≥ 90 mmHg), high blood sugar (HBS)/diabetes (defined by a history of HBS/diabetes, or fasting blood glucose ≥ 6.1mmol/L, or non-fasting blood glucose ≥ 7.8mmol/L), a history of cancer, cardiovascular disease, stroke, asthma, lung disease, liver disease, digestive disease, kidney disease, arthritis, memory problem and psychological problem.

All-cause mortality follow-up

Participants enrolled in wave 1 were followed up in subsequent three waves. In wave 2, both the interview status (dead or alive) and death time were recorded. In waves 3 and 4, only the interview status was recorded. For those who had the exact time on all-cause death in wave 2, the survival time was defined as the interval between the interview time of wave 1 and the death time. If the exact death time was not available in waves 3 and 4, the survival time was computed as the median of the interval between wave 1 and the specific wave with death information. For those who did not die during the follow-up period, the survival time was defined as the interval between wave 1 and the last interview wave with follow-up information.

Patient and public involvement

Anonymised participant data were used in this study. Patients and the public were not involved in the design or conduct, or reporting, or dissemination plans of the study.

Statistical analysis

Data were presented as median (P_{25}~P_{75}) for continuous variables and frequency (percentage) for categorical variables. Baseline characteristics between or among groups were compared by the Wilcoxon rank sum test or Kruskal-Wallis rank sum test for continuous variables and by the chi-square test for categorical variables. The Cox proportional hazard ratio model was used to estimate the hazard ratios (HRs) and

For peer review only - http://bmjopen.bmj.com/site/about/guidelines.xhtml

95% confidence intervals (CIs) of LDL-C quintiles. In addition, the association between all-cause mortality and LDL-C on a continuous scale was further examined using restricted cubic splines (RCS) incorporated in Cox proportional hazards models. Bayesian Information Criterion (BIC) was used to determine the optimal number of knots in RCS. In this study, 3 knots were used in all RCS analyses, with knot locations at the 10th, 50th, and 90th percentiles of LDL-C. To be consistent with quintile analyses, the reference point was the 20th percentile of LDL-C in both men and women. All statistical analyses were performed by SAS statistical software (version 9.4, Cary, NC). All *P* values were 2-tailed, and the significance level was set at 0.05.

Results

Baseline characteristics of the study population

A total of 4981 men and 5529 women were eligible for the final analysis. The median of LDL-C levels in women was significantly higher than that in men ($P < 0.0001$, Supplementary Figure S1). Compared with women, men were older, had smaller BMI values and possessed greater smoking rate and drinking rate (all $P < 0.0001$). The prevalence rates of heart disease, arthritis and digestive disease in women were higher than those in men, but the prevalence rates of asthma and lung disease were lower in women (all $P < 0.0001$, Table 1).

Characteristics of men and women according to the quintiles of LDL-C levels

After stratification by the quintiles of LDL-C levels, BMI, SBP, DBP and hemoglobin in men were elevated with ascending quintiles as a whole (All P<0.001) (Table 2). The prevalence of HBS/diabetes was highest in the bottom quintile of LDL-C and lowest in the fourth quintile, with prevalence rates of 34.81% and 27.06% respectively. There were no statistical differences among LDL-C quintiles for many other characteristics (e.g. age, smoking, drinking, ADL disability, living alone, stroke, cancer, heart disease, lung disease, liver disease, kidney disease, digestive disease, asthma, arthritis, psychological problem and memory problem) (All P> 0.05).

In women, LDL-C quintiles were positively associated with age, BMI, SBP,

For peer review only - http://bmjopen.bmj.com/site/about/guidelines.xhtml

DBP and hemoglobin (All P <0.001) (Table 3). The prevalence rates of HBS/diabetes and liver disease in women were significantly different among different LDL-C quintiles (All P<0.001). For the remaining variables (e.g., smoking, drinking, household income, ADL disability, educational level, rural residence, stroke, cancer, heart disease, lung disease, kidney disease, digestive disease, asthma, arthritis, psychological problem and memory problem), no differences were observed (All P > 0.05).

Associations of LDL-C levels with all-cause mortality

In men, 305 out of 4981 participants died during a four-year follow-up. The mortality rates were declining with ascending quintiles (Table 4). Compared with the first quintile, the univariate HRs (95%CIs) were 0.733(0.533~1.007) for the second quintile; 0.639(0.458~0.892) for the third quintile; 0.519(0.364~0.739) for the fourth quintile and 0.512(0.359~0.732) for the fifth quintile. After adjustment for a series of potential confounders, the non-linear association between LDL-C and all-cause mortality was observed. As compared with the first quintile, the multivariable HRs (95%CIs) were as follows: second quintile, 0.866(0.567~1.325); third quintile, 0.782(0.507~1.206); fourth quintile, 0.577(0.363~0.916); fifth quintile, 0.788(0.497~1.248). In women, there were 219 deaths during a four-year follow-up. The mortality rate was highest in the first quintile. After adjustment for potential confounders, no quintile showed significant lower mortality rates compared with the first quintile (all P> 0.05) (Table 4).

The quintile analysis indicated that the relationship between LDL-C with all-cause mortality might be non-linear. Therefore, RCS was further performed to investigate the association between all-cause mortality and LDL-C on a continuous scale. The results from RCS showed that when the 20th percentile of LDL-C levels was used as the reference, lower LDL-C was associated with higher risk of 4-year all-cause mortality in men, and moderately higher LDL-C possessed lower total mortality risk, but the association was not statistically significant for much higher LDL-C concentrations (Figure 2). The sub-group analyses by age indicated that when the 20th percentile of LDL-C levels was taken as the reference, a lower level of

For peer review only - http://bmjopen.bmj.com/site/about/guidelines.xhtml

LDL-C was associated with a higher risk of 4-year all-cause mortality in both middle-aged (45~60 years) and elderly (≥60 years) men (Figure 3). For women, LDL-C was not significantly associated with 4-year all-cause mortality (Figures 2 and 3).

Discussion

In this study, we investigated the relationship between LDL-C and 4-year all-cause mortality among the middle-aged and elderly Chinese population. In men, a very low level of LDL-C was associated with increased mortality risk. In women, LDL-C was not significantly associated with 4-year all-cause mortality.

Low-density lipoprotein has been well established as an important cause of cardiovascular disease (CVD) for decades[1]. Since CVD is the leading cause of mortality throughout the world, it is logically reasonable that increased LDL-C should contribute to increased CVD mortality and possibly all-cause mortality. Indeed, evidences from prospective epidemiologic studies showed a positive association between non-HDL-C concentration and ischaemic heart disease mortality[8]. However, non-HDL-C includes both LDL-C and very low-density lipoprotein cholesterol (VLDL-C). It is surprising that the direct association of LDL-C with CVD mortality was not consistently reported among studies. Abdullah *et al.* (2018) demonstrated that LDL-C was independently associated with CVD mortality in a low 10-year risk cohort with long-term follow-up[9]. By contrast, Tikhonof *et al.* (2005) reported that the elderly subjects (≥ 65 years) possessed the highest CVD mortality in the lowest LDL-C quartile[10]. Meanwhile, many other studies also found no association between LDL-C and CVD mortality[11-13]. When the results about the association of LDL-C with CVD mortality were inconsistent, it is more surprising to find that few studies have reported the positive association between LDL-C and all-cause mortality. In the study by Abdullah *et al.* (2018), there were already no associations or minimal positive associations between high LDL-C categories and all-cause mortality even in univariable Cox analyses[9]. Most remarkably, results of multivariable Cox analyses in this study were not provided for all-cause mortality,

For peer review only - http://bmjopen.bmj.com/site/about/guidelines.xhtml

which could exert a substantial impact on the final association[14]. Actually, a large number of studies reported no association or even an inverse association between LDL-C and all-cause mortality, which has been summarized in a systematic review by Ravnskov *et al.* (2016)[5]. Therefore, although the mainstream view has been advocating the benefit of lowering high LDL-C, the harmful effect of very low LDL-C may be largely neglected.

It should be noted that the confounding effect of statin treatment should be minimized, as this study excluded those who used lipid-lowering interventions. There was also no association between baseline LDL-C and the presence of cancer, stroke and heart disease in men and women (Tables 2 and 3). Moreover, when participants who had died during the first observation year were excluded, this relationship was not changed (Supplementary Table S1 and Figure S2). This could relieve the concern that serious diseases may lower cholesterol soon before death occurs. One of the possible reasons for the difference between men and women may be due to fewer death events in women than in men, which might result in insufficient power for the association.

Several explanations for the unfavorable effects of low LDL-C levels may be proposed. LDL-C has been suggested to play an important role in host defense against both bacterial and viral pathogens[15]. Indeed, many animal and laboratory experiments have shown that LDL could bind to and inactivate a broad range of microorganisms and their toxic products[16-18]. This hypothesis may be further supported by the recent finding that LDL-C was associated with reduced infectious mortality based on the data from 37,250 patients in the international Monitoring Dialysis Outcomes (MONDO) database[2]. In addition, it has been proposed that LDL-C may have the potential to protect against cancer as many cancer types are caused by viruses[19]. Ravnskov *et al.* (2012) reviewed nine cohort studies including more than 140,000 individuals followed for 10–30 years and found that low cholesterol was associated with cancer[20]. Moreover, cholesterol-lowering experiments on rodents have led to cancer as well[21]. In agreement with these findings, individuals with familial hypercholesterolaemia have been found to possess

For peer review only - http://bmjopen.bmj.com/site/about/guidelines.xhtml

significantly lower cancer mortality[22]. Therefore, lower LDL-C may contribute to a higher risk of death from infection and cancer, which in turn results in increased all-cause mortality.

This study demonstrated that middle-aged and elderly Chinese men with low LDL-C had an increased risk of all-cause mortality, which calls for special attention to be paid to the possible harmful effect of a very low level of LDL-C. However, some limitations should be noted. First, the follow-up period was limited to 4 years. For a longer follow-up time, the associations between LDL-C and all-cause mortality in women might be displayed. In addition, cause-specific mortality data were not available for the time being, preventing the analysis of the association between LDL-C and cause-specific mortality. Moreover, there are issues of multiple testing for comparisons of characteristics among LDL-C quintiles, which could result in Type I error inflation. At last, some of the measured co-morbidities were not specified and detailed in the database, such as lung disease, digestive disease, liver disease, kidney disease, psychological problem and memory problem.

In China, 4-year total mortality is associated with a very low level of plasma LDL-C in middle-aged and elderly men. The findings in this study may suggest the potential harmful effect of a quite low level of LDL-C. More prospective and well-designed studies are needed to validate the relationship between LDL-C and mortality.

Acknowledgements

This analysis uses data or information from the Harmonized CHARLS dataset and Codebook, Version C as of April 2018 developed by the Gateway to Global Aging Data. The development of the Harmonized CHARLS was funded by the National Institute on Ageing (R01 AG030153, RC2 AG036619, R03 AG043052). For more information, please refer to www.g2aging.org.

Funding

This work was supported by National Natural Science Foundation of China

For peer review only - http://bmjopen.bmj.com/site/about/guidelines.xhtml

(81703316 to C.K., 81703322 to Y.W.) and Natural Science Foundation of Jiangsu Province (BK20170350).

Competing interests

None declared.

Patient consent for publication

Obtained

Contributors

CK and YS conceived and designed the research; LZ and CK wrote the manuscript; and YW and SY performed the data analysis. All authors contributed to the interpretations of the findings. All authors reviewed the manuscript.

Ethics approval

CHARLS was approved by Biomedical Ethics Review Committee of Peking University, and all participants signed informed consents.

Data sharing statement

The data used and analysed in this study are publicly available from the China Health and Retirement Longitudinal Study (http://charls.pku.edu.cn/zh-CN).

Figure legends

Figure 1 Flowchart on the selection of eligible participants.

Figure 2 Results from restricted cubic splines for the association between LDL-C and 4-year all-cause mortality in men and women, respectively. The multivariable models were adjusted for age, smoking, drinking, BMI, marital status, household income, educational level, rural residence, ADL disability, HDL-C, triglyceride, hemoglobin, hypertension, HBS/diabetes, history of stroke, cancer, heart disease, lung disease,

For peer review only

For peer review only - http://bmjopen.bmj.com/site/about/guidelines.xhtml

liver disease, kidney disease, digestive disease, asthma, arthritis, psychological problem and memory problem.

Figure 3 Results from restricted cubic spline for the association between LDL-C and 4-year all-cause mortality for middle-aged (45~60 years old) and elderly (≥ 60 years old) people, respectively. The multivariable models were adjusted for age, smoking, drinking, BMI, marital status, household income, educational level, rural residence, ADL disability, HDL-C, triglyceride, hemoglobin, hypertension, HBS/diabetes, history of stroke, cancer, heart disease, lung disease, liver disease, kidney disease, digestive disease, asthma, arthritis, psychological problem and memory problem.

References

1. Ference BA, Ginsberg HN, Graham I, et al. Low-density lipoproteins cause atherosclerotic cardiovascular disease. 1. Evidence from genetic, epidemiologic, and clinical studies. A consensus statement from the European Atherosclerosis Society Consensus Panel. Eur Heart J 2017;**38**(32):2459-72 |.
2. Kaysen GA, Ye X, Raimann JG, et al. Lipid levels are inversely associated with infectious and all-cause mortality: international MONDO study results. Journal of lipid research 2018;**59**(8):1519-28|.
3. Chang JJ, Katsanos AH, Khorchid Y, et al. Higher low-density lipoprotein cholesterol levels are associated with decreased mortality in patients with intracerebral hemorrhage. Atherosclerosis 2018;**269**:14-20|.
4. Charach G, George J, Roth A, et al. Baseline low-density lipoprotein cholesterol levels and outcome in patients with heart failure. The American journal of cardiology 2010;**105**(1):100-4|.
5. Ravnskov U, Diamond DM, Hama R, et al. Lack of an association or an inverse association between low-density-lipoprotein cholesterol and mortality in the elderly: a systematic review. Bmj Open 2016;**6**(6): e010401.
6. Heart Protection Study Collaborative G. MRC/BHF Heart Protection Study of cholesterol lowering with simvastatin in 20,536 high-risk individuals: a randomised placebo-controlled trial. Lancet 2002;**360**(9326):7-22|.
7. Zhao Y, Hu Y, Smith JP, et al. Cohort profile: the China Health and Retirement Longitudinal Study (CHARLS). Int J Epidemiol 2014;**43**(1):61-8|.
8. Prospective Studies C, Lewington S, Whitlock G, et al. Blood cholesterol and vascular mortality by age, sex, and blood pressure: a meta-analysis of individual data from 61 prospective studies with 55,000 vascular deaths. Lancet 2007;**370**(9602):1829-39|.
9. Abdullah SM, Defina LF, Leonard D, et al. Long-Term Association of Low-Density Lipoprotein Cholesterol With Cardiovascular Mortality in Individuals at Low 10-Year Risk of Atherosclerotic Cardiovascular Disease. Circulation 2018;**138**(21):2315-25|.
10. Tikhonoff V, Casiglia E, Mazza A, et al. Low-density lipoprotein cholesterol and mortality in older people. Journal of the American Geriatrics Society 2005;**53**(12):2159-64|.

For peer review only - http://bmjopen.bmj.com/site/about/guidelines.xhtml

11. Raiha I, Marniemi J, Puukka P, et al. Effect of serum lipids, lipoproteins, and apolipoproteins on vascular and nonvascular mortality in the elderly. Arteriosclerosis, thrombosis, and vascular biology 1997;**17**(7):1224-32.

12. Upmeier E, Lavonius S, Lehtonen A, et al. Serum lipids and their association with mortality in the elderly: a prospective cohort study. Aging Clin Exp Res 2009;**21**(6):424-30|.

13. Blekkenhorst LC, Prince RL, Hodgson JM, et al. Dietary saturated fat intake and atherosclerotic vascular disease mortality in elderly women: a prospective cohort study. The American journal of clinical nutrition 2015;**101**(6):1263-8|.

14. Ke CF, Shen YP. Letter by Ke and Shen Regarding Article, "Long-Term Association of Low-Density Lipoprotein Cholesterol With Cardiovascular Mortality in Individuals at Low 10-Year Risk of Atherosclerotic Cardiovascular Disease: Results From the Cooper Center Longitudinal Study". Circulation 2019;**139**(18):2190-91|.

15. Feingold KR, Grunfeld C. Lipids: a key player in the battle between the host and microorganisms. Journal of lipid research 2012;**53**(12):2487-89|.

16. Han RL. Plasma lipoproteins are important components of the immune system. Microbiol Immunol 2010;**54**(4):246-53|.

17. Khovidhunkit W, Kim MS, Memon RA, et al. Effects of infection and inflammation on lipid and lipoprotein metabolism: mechanisms and consequences to the host. Journal of lipid research 2004;**45**(7):1169-96|.

18. Ravnskov U, McCully KS. Review and Hypothesis: Vulnerable plaque formation from obstruction of Vasa vasorum by homocysteinylated and oxidized lipoprotein aggregates complexed with microbial remnants and LDL autoantibodies. Annals of clinical and laboratory science 2009;**39**(1):3-16.

19. Read SA, Douglas MW. Virus induced inflammation and cancer development. Cancer letters 2014;**345**(2):174-81|.

20. Ravnskov U, McCully KS, Rosch PJ. The statin-low cholesterol-cancer conundrum. QJM : monthly journal of the Association of Physicians 2012;**105**(4):383-8.

21. Newman TB, Hulley SB. Carcinogenicity of lipid-lowering drugs. Jama 1996;**275**(1):55-60.

22. Neil HA, Hawkins MM, Durrington PN, et al. Non-coronary heart disease mortality and risk of fatal cancer in patients with treated heterozygous familial hypercholesterolaemia: a prospective registry study. Atherosclerosis 2005;**179**(2):293-7.

For peer review only

For peer review only - http://bmjopen.bmj.com/site/about/guidelines.xhtml

Table 1 Characteristics of the study population

Characteristics	Men (n=4981)	Women (n=5529)	P value
Age-yr	59 (53~66)	57 (51~65)	<0.0001
BMI-kg/m^2	22.40 (20.35~24.83)	23.51 (21.17~26.14)	<0.0001
SBP-mmHg	127.67 (115.67~141.67)	127.00 (114.00~143.33)	0.3764
DBP-mmHg	75.33 (67.67~83.67)	74.33 (67.00~82.67)	0.0017
Above-average household income-no. (%)	2104 (49.68)	2360 (50.32)	0.5468
Education level-no. (%)			<0.0001
1	4287 (86.07)	5134 (92.86)	
2	592 (11.89)	347 (6.28)	
3	102 (2.05)	48 (0.87)	
ADL disability-no. (%)	716 (14.55)	1011 (18.57)	<0.0001
Living alone-no. (%)	471 (9.46)	832 (15.05)	<0.0001
Rural residence-no. (%)	3282 (65.89)	3524 (63.74)	0.0210
Lifestyle-no. (%)			
Smoking ever	3738 (75.24)	420 (7.63)	<0.0001
Drinking ever	3297 (66.43)	826 (15.01)	<0.0001
Disease history-no. (%)			
Hypertension	1904 (43.35)	2245 (45.34)	0.0530
HBS/Diabetes	1460 (30.16)	1564 (29.19)	0.2846
Cancer	40 (0.81)	67 (1.22)	0.0372
Stroke	133 (2.69)	136 (2.48)	0.4958
Heart disease	499 (10.10)	738 (13.47)	<0.0001
Lung disease	620 (12.54)	508 (9.26)	<0.0001
Arthritis	1548 (31.23)	2243 (40.80)	<0.0001
Liver disease	197 (4.00)	188 (3.44)	0.1326
Kidney disease	309 (6.27)	315 (5.75)	0.2701
Digestive disease	1030 (20.79)	1393 (25.37)	<0.0001
Asthma	283 (5.72)	216 (3.93)	<0.0001
Psychological problem	60 (1.21)	94 (1.72)	0.0336
Memory problem	97 (1.96)	86 (1.57)	0.1238
Laboratory measurements			
LDL cholesterol-mg/dL	109.41 (88.92~131.06)	117.91 (96.26~141.50)	<0.0001
Triglyceride-mg/dL	96.46 (69.03~145.14)	110.63 (79.65~159.30)	<0.0001
HDL cholesterol-mg/dL	48.71 (39.43~59.54)	50.64 (41.75~60.31)	<0.0001
Hemoglobin-(g/dL)	15.10 (14.00~16.20)	13.60 (12.50~14.60)	<0.0001

ADL, activity of daily living; BMI, body mass index; SBP, systolic blood pressure; DBP, diastolic blood pressure; HBS, high blood sugar.

Educational level: 1, Less than lower secondary education; 2, Upper secondary & vocational training; 3, Tertiary education.

For peer review only - http://bmjopen.bmj.com/site/about/guidelines.xhtml

Table 2 Baseline characteristics of participants by quintiles of LDL-C in men

Characteristics	Quintile 1 (*n*=991) (≤83.89mg/dL)	Quintile 2 (*n*=1008) (83.89~101.68)	Quintile 3 (*n*=991) (101.68~117.14)	Quintile 4 (*n*=1004) (117.14~136.86)	Quintile 5 (*n*=987) (>136.86)	*P* value
Age-yr	59 (52~66)	59 (53~67)	59 (52~66)	59 (53~67)	59 (53~65)	0.5405
BMI-kg/m^2	21.57 (19.78~24.21)	22.18 (20.31~24.40)	22.36 (20.44~24.61)	22.64 (20.65~25.07)	23.27 (20.95~25.68)	<0.0001
SBP-mmHg	126.00 (114.00~140.00)	126.00 (115.33~139.67)	127.00 (114.67~142.33)	128.00 (115.67~142.50)	130.67 (118.00~144.00)	0.0002
DBP-mmHg	75.00 (66.67~83.33)	74.33 (66.67~82.00)	74.67 (67.33~83.33)	75.33 (67.67~83.67)	76.67 (69.33~85.33)	0.0002
Above-average household income-no. (%)	402 (48.43)	413 (47.91)	407 (48.05)	430 (49.20)	452 (54.99)	0.0186
Educational level-no. (%)						0.0035
1	870 (87.79)	891 (88.39)	821 (82.85)	862 (85.86)	843 (85.41)	
2	105 (10.60)	94 (9.33)	145 (14.63)	123 (12.25)	125 (12.66)	
3	16 (1.61)	23 (2.28)	25 (2.52)	19 (1.93)	19 (1.93)	
ADL disability-no. (%)	149 (15.27)	163 (16.30)	148 (15.10)	141 (14.23)	115 (11.81)	0.0590
Living alone-no. (%)	101 (10.19)	112 (11.11)	85 (8.58)	94 (9.36)	79 (8.00)	0.1264
Rural residence-no. (%)	659 (66.50)	702 (69.64)	634 (63.98)	664 (66.14)	623 (63.12)	0.0216
Lifestyle-no. (%)						
Smoking ever	755 (76.57)	754 (75.02)	751 (75.94)	759 (75.75)	719 (72.92)	0.3788
Drinking ever	654 (66.40)	663 (66.10)	664 (67.14)	664 (66.40)	652 (66.13)	0.9890
Disease history-no. (%)						
Hypertension	359 (42.14)	349 (39.48)	385 (42.87)	397 (44.51)	414 (47.81)	0.0092
HBS/Diabetes	337 (34.81)	277 (28.35)	266 (27.85)	266 (27.06)	314 (32.78)	0.0003
Cancer	13 (1.32)	7 (0.70)	5 (0.51)	6 (0.60)	9 (0.92)	0.2728
Stroke	26 (2.64)	33 (3.31)	21 (2.13)	25 (2.50)	28 (2.85)	0.5818
Heart disease	86 (8.78)	98 (9.81)	103 (10.47)	113 (11.31)	99 (10.10)	0.4453
Lung disease	131 (13.37)	135 (13.46)	106 (10.77)	120 (12.00)	128 (13.09)	0.3170
Arthritis	300 (30.43)	332 (33.10)	280 (28.34)	312 (31.23)	324 (33.03)	0.1233
Liver disease	51 (5.23)	42 (4.19)	35 (3.57)	38 (3.81)	31 (3.18)	0.1855
Kidney disease	56 (5.73)	73 (7.29)	59 (6.02)	57 (5.71)	64 (6.56)	0.5518
Digestive disease	211 (21.46)	213 (21.24)	225 (22.82)	203 (20.28)	178 (18.14)	0.1264
Asthma	66 (6.76)	49 (4.89)	43 (4.36)	59 (5.91)	66 (6.72)	0.0754
Psychological problem	14 (1.43)	10 (1.00)	14 (1.42)	14 (1.40)	8 (0.82)	0.6092
Memory problem	25 (2.55)	13 (1.30)	21 (2.13)	19 (1.90)	19 (1.94)	0.3810
Laboratory measurements						
LDL cholesterol-mg/dL	71.91 (61.47~78.09)	93.56 (88.92~97.81)	109.41 (105.54~113.27)	126.42 (121.39~131.44)	153.87 (144.20~170.49)	<0.0001
Triglyceride-mg/dL	96.46 (65.49~177.88)	88.50 (64.61~129.21)	92.93 (69.03~138.95)	97.35 (72.57~136.29)	108.86 (79.65~ 152.22)	<0.0001
HDL cholesterol-mg/dL	47.55	49.48	47.93	49.10	48.71	0.0061

For peer review only - http://bmjopen.bmj.com/site/about/guidelines.xhtml

	(35.95~59.92)	(39.43~60.70)	(39.43~58.76)	(39.82~59.54)	(41.37~58.76)	
Hemoglobin-(g/dL)	14.80 (13.60~16.00)	14.90 (13.80~16.10)	15.00 (14.00~16.20)	15.10 (14.00~16.30)	15.40 (14.30~16.50)	<0.0001

Table 3 Baseline characteristics of participants by quintiles of LDL-C in women

Characteristics	Quintile 1 (*n*=1114) (≤91.24mg/dL)	Quintile 2 (*n*=1102) (91.24~109.41)	Quintile 3 (*n*=1096) (109.41~126.03)	Quintile 4 (*n*=1111) (126.03~147.68)	Quintile 5 (*n*=1106) (>147.68)	*P* value
Age-yr	56 (49~64)	56 (49~64)	57 (50~63)	58 (52~65)	59 (53~66)	<0.0001
BMI-kg/m^2	23.15 (20.90~25.76)	23.30 (21.01~25.82)	23.39 (21.10~26.02)	23.63 (21.32~26.26)	24.12 (21.57~26.83)	<0.0001
SBP-mmHg	124.67 (112.00~141.00)	125.67 (113.00~142.33)	127.00 (114.67~143.00)	127.67 (114.67~142.33)	130.00 (117.00~146.00)	<0.0001
DBP-mmHg	73.33 (65.67~81.67)	74.33 (66.67~82.67)	74.00 (67.33~82.67)	74.67 (67.67~82.67)	75.33 (67.67~83.33)	0.0103
Above-average household income-no. (%)	458 (48.21)	482 (50.42)	461 (48.99)	497 (53.61)	462 (50.44)	0.1720
Education level-no. (%)						0.4079
1	1023 (91.83)	1030 (93.47)	1023 (93.34)	1025 (92.26)	1033 (93.40)	
2	76 (6.82)	67 (6.08)	63 (5.75)	76 (6.84)	65 (5.88)	
3	15 (1.35)	5 (0.45)	10 (0.91)	10 (0.90)	8 (0.72)	
ADL disability-no. (%)	216 (19.69)	183 (16.90)	188 (17.59)	215 (19.58)	209 (19.05)	0.3416
Living alone-no. (%)	155 (13.91)	162 (14.70)	143 (13.05)	186 (16.74)	186 (16.82)	0.0429
Rural residence-no. (%)	715 (64.18)	695 (63.07)	726 (66.24)	706 (63.55)	682 (61.66)	0.2526
Lifestyle-no. (%)						
Smoking ever	71 (6.39)	93 (8.49)	78 (7.15)	79 (7.14)	99 (8.97)	0.1294
Drinking ever	173 (15.61)	160 (14.61)	169 (15.50)	165 (14.91)	159 (14.40)	0.9112
Disease history-no. (%)						
Hypertension	414 (41.69)	440 (44.58)	431 (44.21)	451 (45.46)	509 (50.70)	0.0014
HBS/Diabetes	310 (28.78)	290 (27.46)	280 (26.19)	311 (28.88)	373 (34.53)	0.0003
Cancer	14 (1.27)	15 (1.38)	18 (1.66)	14 (1.27)	6 (0.54)	0.1876
Stroke	31 (2.80)	16 (1.47)	27 (2.47)	31 (2.81)	31 (2.81)	0.1906
Heart disease	144 (13.08)	135 (12.41)	140 (12.89)	166 (15.09)	153 (13.87)	0.3873
Lung disease	110 (9.94)	112 (10.29)	103 (9.45)	91 (8.27)	92 (8.36)	0.3566
Arthritis	454 (41.05)	450 (41.21)	421 (38.55)	454 (41.05)	464 (42.14)	0.5189
Liver disease	53 (4.80)	40 (3.69)	25 (2.31)	43 (3.91)	27 (2.46)	0.0060
Kidney disease	74 (6.71)	62 (5.69)	57 (5.24)	66 (6.02)	56 (5.09)	0.4886
Digestive disease	279 (25.20)	267 (24.45)	269 (24.70)	299 (27.13)	279 (25.34)	0.6323
Asthma	43 (3.89)	43 (3.94)	44 (4.03)	39 (3.54)	47 (4.26)	0.9384
Psychological problem	16 (1.45)	19 (1.74)	23 (2.12)	17 (1.54)	19 (1.73)	0.7943
Memory problem	18 (1.63)	19 (1.74)	11 (1.01)	15 (1.36)	23 (2.09)	0.3245

For peer review only - http://bmjopen.bmj.com/site/about/guidelines.xhtml

Laboratory measurements						
LDL cholesterol-mg/dL	78.48 (68.04~85.83)	100.90 (96.26~105.54)	117.91 (113.66~121.78)	135.70 (130.28~141.50)	165.08 (155.03~179.00)	<0.0001
Triglyceride-mg/dL	110.63 (73.46~192.93)	103.54 (74.34~153.99)	107.97 (77.88~152.22)	110.63 (82.31~151.34)	125.23 (92.93~162.84)	<0.0001
HDL cholesterol-mg/dL	46.39 (35.57~57.60)	50.64 (40.98~60.70)	51.42 (42.53~60.70)	51.80 (43.69~61.08)	51.80 (44.46~60.31)	<0.0001
Hemoglobin-(g/dL)	13.20 (12.10~14.30)	13.30 (12.30~14.50)	13.60 (12.60~14.70)	13.70 (12.80~14.60)	13.80 (12.80~14.80)	<0.0001

Table 4 Associations between all-cause mortality and LDL-C

	Total	Deaths (%)	Unadjusted		Adjusted*	
			HR (95%CI)	*P* value	HR (95%CI)	*P* value
Men						
Q1	991	88(8.88)	1	-	1	-
Q2	1008	67(6.65)	0.733(0.533~1.007)	0.0554	0.866(0.567~1.325)	0.5079
Q3	991	57(5.75)	0.639(0.458~0.892)	0.0084	0.782(0.507~1.206)	0.2651
Q4	1004	47(4.68)	0.519(0.364~0.739)	0.0003	0.577(0.363~0.916)	0.0197
Q5	987	46(4.66)	0.512(0.359~0.732)	0.0002	0.788(0.497~1.248)	0.3093
Women						
Q1	1114	52(4.66)	1	-	1	-
Q2	1102	49(4.45)	0.960(0.650~1.419)	0.8394	1.348(0.816~2.229)	0.2440
Q3	1096	29(2.64)	0.565(0.359~0.890)	0.0138	0.675(0.375~1.214)	0.1889
Q4	1111	41(3.69)	0.792(0.526~1.192)	0.2632	0.974(0.567~1.674)	0.9239
Q5	1106	48(4.34)	0.926(0.625~1.371)	0.7007	1.043(0.620~1.755)	0.8736

*Adjusted for age, smoking, drinking, BMI, marital status, household income, educational level, rural residence, ADL disability, HDL-C, triglyceride, hemoglobin, hypertension, HBS/diabetes, history of stroke, cancer, heart disease, lung disease, liver disease, kidney disease, digestive disease, asthma, arthritis, psychological problem and memory problem.

For peer review only - http://bmjopen.bmj.com/site/about/guidelines.xhtml

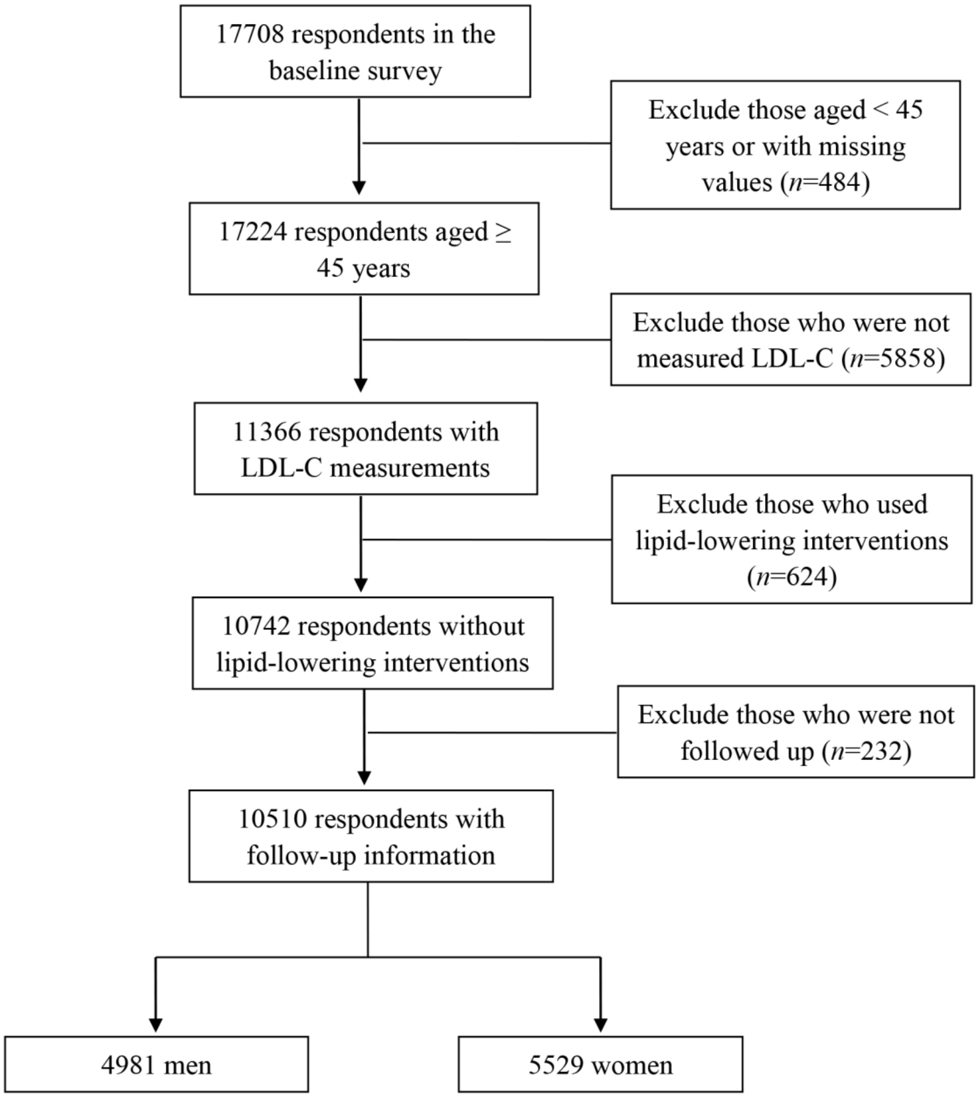

Figure 1 Flowchart on the selection of eligible participants.

151x168mm (300 x 300 DPI)

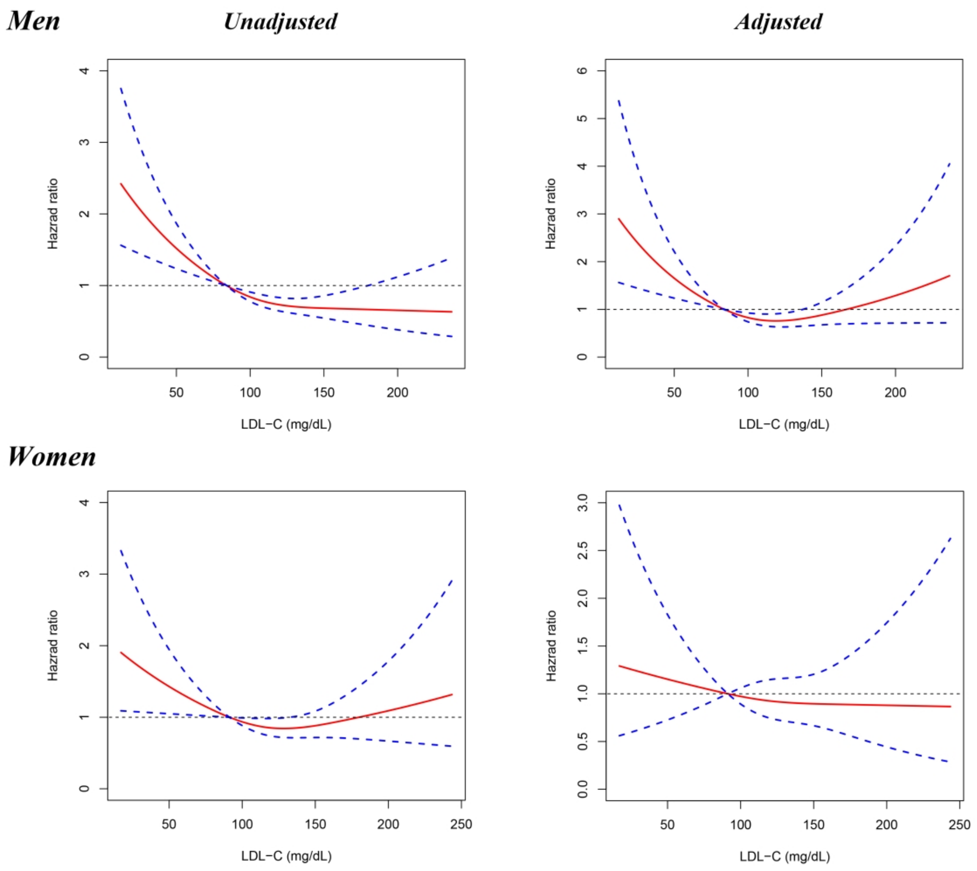

Figure 2 Results from restricted cubic splines for the association between LDL-C and 4-year all-cause mortality in men and women, respectively. The multivariable models were adjusted for age, smoking, drinking, BMI, marital status, household income, educational level, rural residence, ADL disability, HDL-C, triglyceride, hemoglobin, hypertension, HBS/diabetes, history of stroke, cancer, heart disease, lung disease, liver disease, kidney disease, digestive disease, asthma, arthritis, psychological problem and memory problem.

321x289mm (300 x 300 DPI)

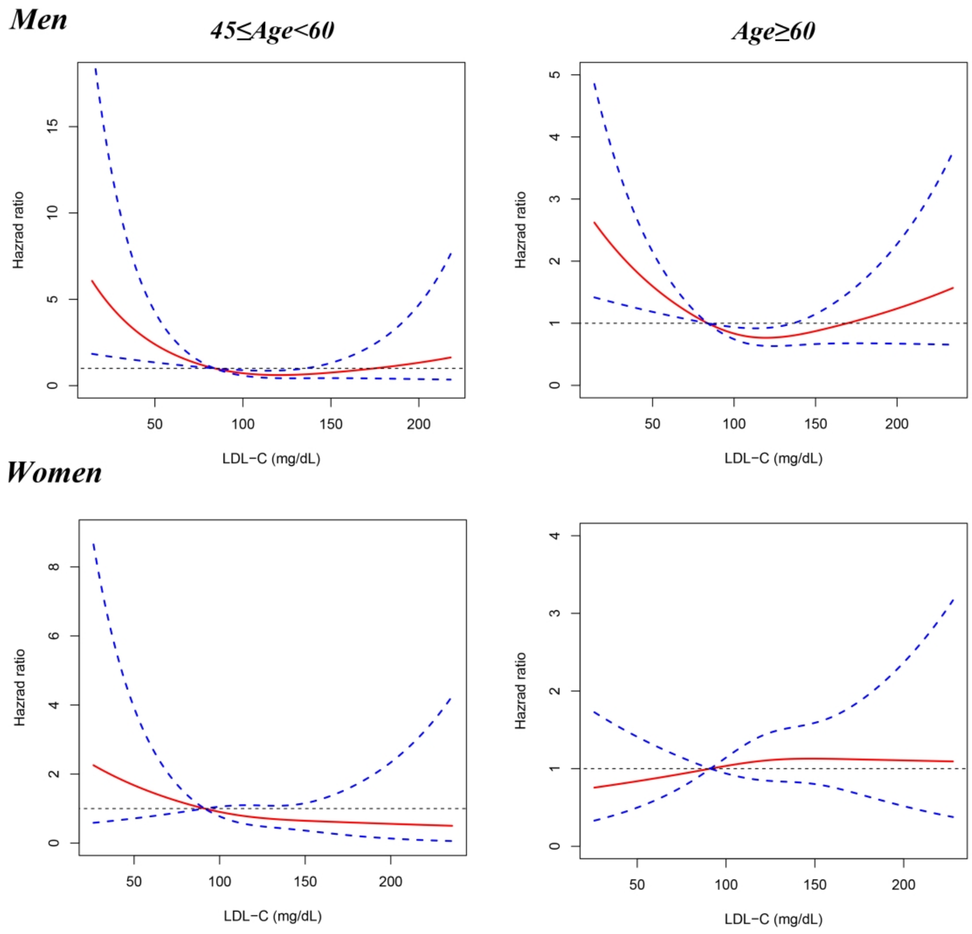

Figure 3 Results from restricted cubic spline for the association between LDL-C and 4-year all-cause mortality for middle-aged (45~60 years old) and elderly (≥ 60 years old) people, respectively. The multivariable models were adjusted for age, smoking, drinking, BMI, marital status, household income, educational level, rural residence, ADL disability, HDL-C, triglyceride, hemoglobin, hypertension, HBS/diabetes, history of stroke, cancer, heart disease, lung disease, liver disease, kidney disease, digestive disease, asthma, arthritis, psychological problem and memory problem.

296x281mm (300 x 300 DPI)

Supplementary materials

Low-density lipoprotein cholesterol and all-cause mortality: findings from the China Health and Retirement Longitudinal Study

Liang Zhou [1], Ying Wu [2], Shaobo Yu [3], Yueping Shen [4] and Chaofu Ke [4]

[1] Liyang Center for Disease Control and Prevention, 55 Nanhuan Road, Liyang 213371, P. R. China.

[2] State Key Laboratory of Organ Failure Research, Department of Biostatistics, Guangdong Provincial Key Laboratory of Tropical Disease Research, School of Public Health, Southern Medical University, Guangzhou 510515, China.

[3] Medical College of Soochow University, Suzhou 215123, P. R. China.

[4] Department of Epidemiology and Biostatistics, School of Public Health, Medical College of Soochow University, 199 Renai Road, Suzhou 215123, P. R. China.

For peer review only

Supplementary Table S1 Associations between all-cause mortality and LDL-C[#]

	Total	Deaths (%)	Unadjusted		Adjusted[*]	
			HR (95%CI)	*P* value	HR (95%CI)	*P* value
Men						
Q1	972	69 (7.10)	1	-	1	-
Q2	999	58 (5.81)	0.806(0.568~1.142)	0.2251	0.978(0.623~1.536)	0.9244
Q3	979	45 (4.60)	0.642(0.441~0.934)	0.0205	0.763(0.473~1.231)	0.2681
Q4	992	35 (3.53)	0.491(0.327~0.737)	0.0006	0.532(0.317~0.893)	0.0169
Q5	982	41 (4.18)	0.579(0.394~0.853)	0.0056	0.819(0.498~1.348)	0.4329
Women						
Q1	1106	44 (3.98)	1	-	1	-
Q2	1094	41 (3.75)	0.951(0.622~1.456)	0.8177	1.284(0.755~2.182)	0.3561
Q3	1094	27 (2.47)	0.622(0.385~1.005)	0.0523	0.702(0.383~1.287)	0.2528
Q4	1105	35 (3.17)	0.799(0.513~1.246)	0.3222	0.912(0.514~1.619)	0.7542
Q5	1099	41 (3.73)	0.934(0.610~1.430)	0.7538	0.995(0.576~1.720)	0.9853

[#]Participants who died during the first year were excluded.

[*]Adjusted for age, smoking, drinking, BMI, living alone, household income, educational level, rural residence, ADL disability, HDL-C, triglyceride, hemoglobin, hypertension, HBS/diabetes, history of stroke, cancer, heart disease, lung disease, liver disease, kidney disease, digestive disease, asthma, arthritis, psychological problem and memory problem.

For peer review only

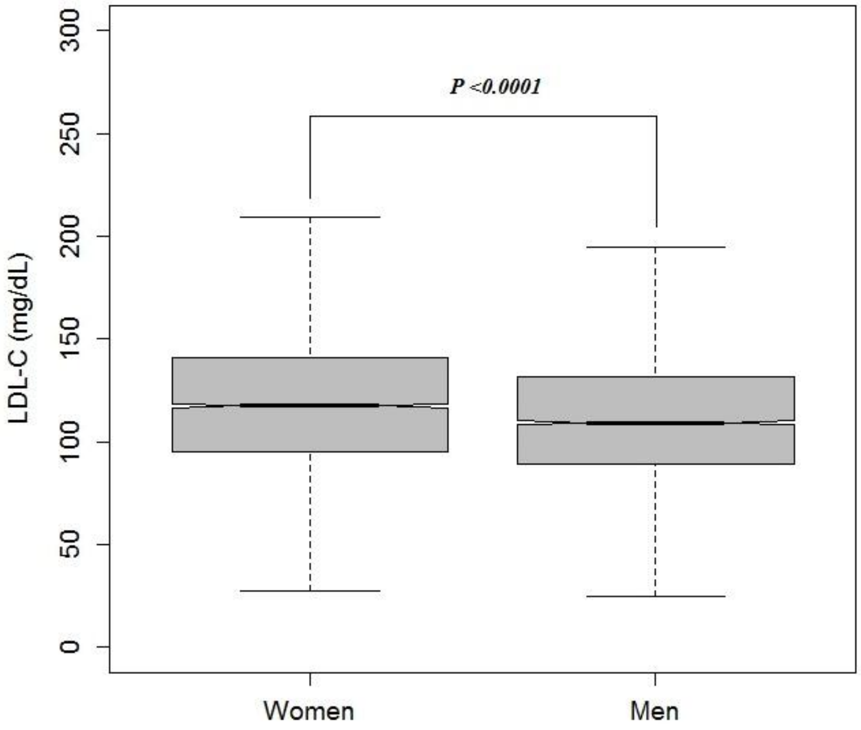

Supplementary Figure S1 The box-plot of plasma LDL-C levels in middle-aged and elderly Chinese men and women.

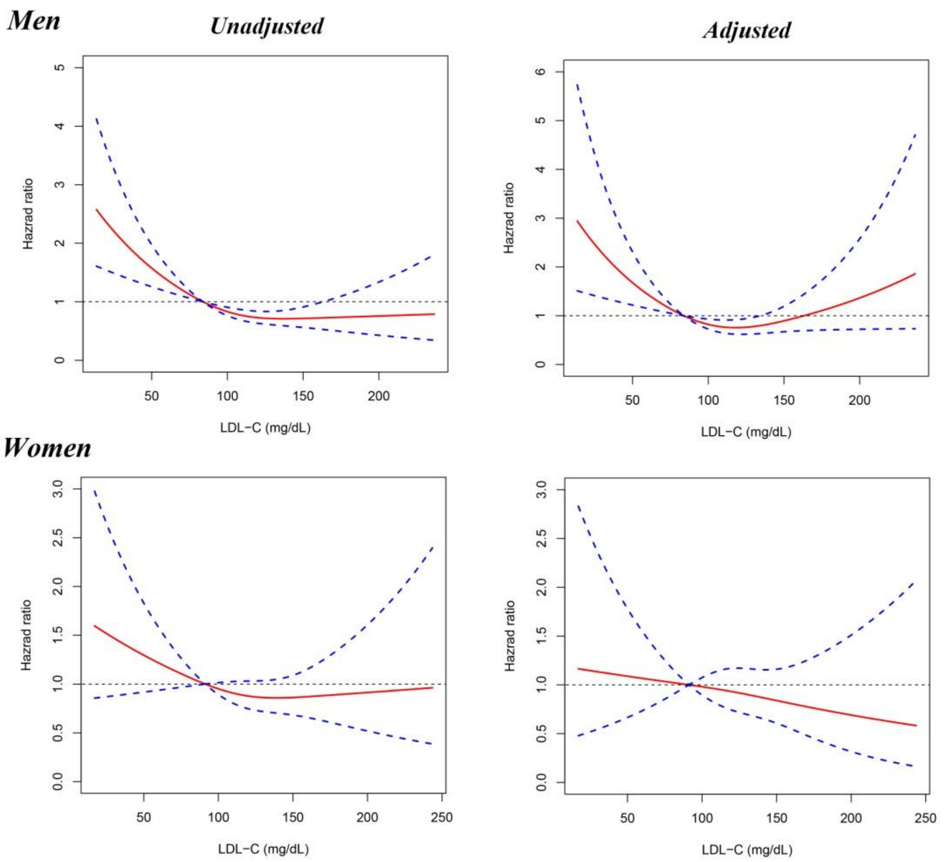

Supplementary Figure S2 Results from restricted cubic splines for the association between LDL-C and 4-year all-cause mortality in men and women (excluding participants who died during the first year). The multivariable models were adjusted for age, smoking, drinking, BMI, living alone, household income, educational level, rural residence, ADL disability, HDL-C, triglyceride, hemoglobin, hypertension, HBS/diabetes, history of stroke, cancer, heart disease, lung disease, liver disease, kidney disease, digestive disease, asthma, arthritis, psychological problem and memory problem.

STROBE 2007 (v4) Statement—Checklist of items that should be included in reports of *cohort studies*

Section/Topic	Item #	Recommendation	Reported on page #
**Title and abstract**	1	(*a*) Indicate the study's design with a commonly used term in the title or the abstract	2
		(*b*) Provide in the abstract an informative and balanced summary of what was done and what was found	2
**Introduction**			
Background/rationale	2	Explain the scientific background and rationale for the investigation being reported	3
Objectives	3	State specific objectives, including any prespecified hypotheses	4
**Methods**			
Study design	4	Present key elements of study design early in the paper	4
Setting	5	Describe the setting, locations, and relevant dates, including periods of recruitment, exposure, follow-up, and data collection	4,5
Participants	6	(*a*) Give the eligibility criteria, and the sources and methods of selection of participants. Describe methods of follow-up	4,5
		(*b*) For matched studies, give matching criteria and number of exposed and unexposed	Not applicable
Variables	7	Clearly define all outcomes, exposures, predictors, potential confounders, and effect modifiers. Give diagnostic criteria, if applicable	4,5
Data sources/ measurement	8*	For each variable of interest, give sources of data and details of methods of assessment (measurement). Describe comparability of assessment methods if there is more than one group	*4,5*
Bias	9	Describe any efforts to address potential sources of bias	4,7,8,9
Study size	10	Explain how the study size was arrived at	4
Quantitative variables	11	Explain how quantitative variables were handled in the analyses. If applicable, describe which groupings were chosen and why	5
Statistical methods	12	(*a*) Describe all statistical methods, including those used to control for confounding	5,6
		(*b*) Describe any methods used to examine subgroups and interactions	5,6,7
		(*c*) Explain how missing data were addressed	4,5
		(*d*) If applicable, explain how loss to follow-up was addressed	5
		(*e*) Describe any sensitivity analyses	4,7,8,9
**Results**			

For peer review only

Participants	13*	(a) Report numbers of individuals at each stage of study—eg numbers potentially eligible, examined for eligibility, confirmed eligible, included in the study, completing follow-up, and analysed	4,5
		(b) Give reasons for non-participation at each stage	4,5
		(c) Consider use of a flow diagram	4
Descriptive data	14*	(a) Give characteristics of study participants (eg demographic, clinical, social) and information on exposures and potential confounders	6
		(b) Indicate number of participants with missing data for each variable of interest	4
		(c) Summarise follow-up time (eg, average and total amount)	5
Outcome data	15*	Report numbers of outcome events or summary measures over time	7
Main results	16	(*a*) Give unadjusted estimates and, if applicable, confounder-adjusted estimates and their precision (eg, 95% confidence interval). Make clear which confounders were adjusted for and why they were included	6,7
		(*b*) Report category boundaries when continuous variables were categorized	5,6,7
		(*c*) If relevant, consider translating estimates of relative risk into absolute risk for a meaningful time period	Not applicable
Other analyses	17	Report other analyses done—eg analyses of subgroups and interactions, and sensitivity analyses	6,7,8,9
**Discussion**			
Key results	18	Summarise key results with reference to study objectives	8
**Limitations**			
Interpretation	20	Give a cautious overall interpretation of results considering objectives, limitations, multiplicity of analyses, results from similar studies, and other relevant evidence	8,9,10
Generalisability	21	Discuss the generalisability (external validity) of the study results	8,9,10
**Other information**			
Funding	22	Give the source of funding and the role of the funders for the present study and, if applicable, for the original study on which the present article is based	10,11

*Give information separately for cases and controls in case-control studies and, if applicable, for exposed and unexposed groups in cohort and cross-sectional studies.

Note: An Explanation and Elaboration article discusses each checklist item and gives methodological background and published examples of transparent reporting. The STROBE checklist is best used in conjunction with this article (freely available on the Web sites of PLoS Medicine at http://www.plosmedicine.org/, Annals of Internal Medicine at http://www.annals.org/, and Epidemiology at http://www.epidem.com/). Information on the STROBE Initiative is available at www.strobe-statement.org.

For peer review only

Low-density lipoprotein cholesterol and all-cause mortality: findings from the China Health and Retirement Longitudinal Study

Journal:	*BMJ Open*
Manuscript ID	bmjopen-2020-036976.R2
Article Type:	Original research
Date Submitted by the Author:	03-Jun-2020
Complete List of Authors:	Zhou, Liang; Liyang Center for Disease Control and Prevention<br>Wu, Ying; Southern Medical University, State Key Laboratory of Organ Failure Research, Department of Biostatistics, Guangdong Provincial Key Laboratory of Tropical Disease Research, School of Public Health<br>Yu, Shaobo; Soochow University Medical College<br>shen, yueping; Soochow University Medical College, Department of Epidemiology and Biostatistics<br>Ke, Chaofu; Soochow University Medical College, Department of Epidemiology and Biostatistics
**Primary Subject Heading**:	Epidemiology
Secondary Subject Heading:	Epidemiology, Public health
Keywords:	Risk management < HEALTH SERVICES ADMINISTRATION & MANAGEMENT, PREVENTIVE MEDICINE, PUBLIC HEALTH, EPIDEMIOLOGY

SCHOLARONE™ Manuscripts

I, the Submitting Author has the right to grant and does grant on behalf of all authors of the Work (as defined in the below author licence), an exclusive licence and/or a non-exclusive licence for contributions from authors who are: i) UK Crown employees; ii) where BMJ has agreed a CC-BY licence shall apply, and/or iii) in accordance with the terms applicable for US Federal Government officers or employees acting as part of their official duties; on a worldwide, perpetual, irrevocable, royalty-free basis to BMJ Publishing Group Ltd ("BMJ") its licensees and where the relevant Journal is co-owned by BMJ to the co-owners of the Journal, to publish the Work in this journal and any other BMJ products and to exploit all rights, as set out in our licence.

The Submitting Author accepts and understands that any supply made under these terms is made by BMJ to the Submitting Author unless you are acting as an employee on behalf of your employer or a postgraduate student of an affiliated institution which is paying any applicable article publishing charge ("APC") for Open Access articles. Where the Submitting Author wishes to make the Work available on an Open Access basis (and intends to pay the relevant APC), the terms of reuse of such Open Access shall be governed by a Creative Commons licence – details of these licences and which Creative Commons licence will apply to this Work are set out in our licence referred to above.

Other than as permitted in any relevant BMJ Author's Self Archiving Policies, I confirm this Work has not been accepted for publication elsewhere, is not being considered for publication elsewhere and does not duplicate material already published. I confirm all authors consent to publication of this Work and authorise the granting of this licence.

For peer review only

Low-density lipoprotein cholesterol and all-cause mortality: findings from the China Health and Retirement Longitudinal Study

Liang Zhou [1†], Ying Wu [2†], Shaobo Yu [3], Yueping Shen [4*] and Chaofu Ke [4*]

[1] Liyang Center for Disease Control and Prevention, 55 Nanhuan Road, Liyang 213371, P. R. China.

[2] State Key Laboratory of Organ Failure Research, Department of Biostatistics, Guangdong Provincial Key Laboratory of Tropical Disease Research, School of Public Health, Southern Medical University, Guangzhou 510515, P. R. China.

[3] Medical College of Soochow University, 199 Renai Road, Suzhou 215123, P. R. China.

[4] Department of Epidemiology and Biostatistics, School of Public Health, Medical College of Soochow University, 199 Renai Road, Suzhou 215123, P. R. China.

[†] Liang Zhou and Ying Wu made equal contributions to this work.

[*]Correspondence should be addressed to:

Chaofu Ke, Ph.D.

Tel.: +86-512-6588-0079

Fax: +86-512-6588-3323

E-mail: cfke@suda.edu.cn

Department of Epidemiology and Biostatistics, School of Public Health, Medical College of Soochow University, 199 Renai Road, Suzhou 215123, P. R. China.

or Yueping Shen, Ph.D.

Tel.: +86-512-6588-0079

Fax: +86-512-6588-3323

E-mail: shenyueping@suda.edu.cn

Department of Epidemiology and Biostatistics, School of Public Health, Medical College of Soochow University, 199 Renai Road, Suzhou 215123, P. R. China.

For peer review only

For peer review only - http://bmjopen.bmj.com/site/about/guidelines.xhtml

Abstract

Objectives

To investigate the relationship between low-density lipoprotein cholesterol (LDL-C) and all-cause mortality among middle-aged and elderly Chinese population.

Design

Prospective cohort study.

Setting

This study used data from the Chinese Health and Retirement Longitudinal Study (CHARLS).

Participants

Middle-aged and elderly participants with complete data were enrolled for a 4-year follow-up of total mortality and plasma levels of LDL-C, including 4981 male respondents and 5529 female respondents.

Results

During a 4-year follow-up, there were 305 and 219 deaths in men and women, respectively. Compared with the first quintile (Q1) of LDL-C, the adjusted hazard ratios (95% confidence intervals) were 0.818(0.531~1.260) for Q2, 0.782(0.507~1.208) for Q3, 0.605(0.381~0.962) for Q4 and 0.803(0.506~1.274) for Q5 in men. The results from restricted cubic spine (RCS) showed that when the 20th percentile of LDL-C levels (84mg/dL) was used as the reference, a lower LDL-C concentration (<84mg/dL) was associated with a higher 4-year all-cause mortality risk. By contrast, both quintile analysis and RCS analysis did not show a statistically significant association in women.

Conclusions

Compared with moderately elevated LDL-C (e.g., 117-137mg/dL), a lower plasma level of LDL-C (e.g., ≤84mg/dL) was associated with an increased risk of 4-year all-cause mortality in middle-aged and elderly Chinese men. The results suggest the potential harmful effect of a quite low level of LDL-C on total mortality.

Keywords Low-density lipoprotein cholesterol, all-cause mortality, CHARLS

For peer review only - http://bmjopen.bmj.com/site/about/guidelines.xhtml

Strengths and limitations of this study

- This study used high-quality data from a nationally representative longitudinal cohort to investigate the relationship between LDL-C and all-cause mortality among middle-aged and elderly Chinese population.
- The use of restricted cubic spline provided a more comprehensive spectrum of the non-linear relation between LDL-C and all-cause mortality.
- Although the public's attention focuses on the benefit of lipid lowering, this study highlights the potential harmful effect of very low LDL-C.
- The 4-year follow-up period prevented the assessment of a long-term association between LDL-C and all-cause mortality.
- The unavailability of cause-specific mortality data prevented the analysis of the association between LDL-C and cause-specific mortality.

Introduction

For decades, the mainstream view holds that a high level of low-density lipoprotein cholesterol (LDL-C) is a primary cause of cardiovascular events and mortality[1]. However, many studies have found that LDL-C levels are inversely associated with all-cause mortality in diseased populations, such as patients with chronic hemodialysis[2], intracerebral hemorrhage[3] and heart failure[4]. Most importantly, in a systematic review of 19 cohort studies including 30 cohorts with 68 094 elderly people (≥60 years), an inverse association between LDL-C and all-cause mortality was seen in 16 cohorts representing 92% of all participants, and none of the other cohorts found the positive association between LDL-C and all-cause mortality[5]. Since LDL-C has been regarded as “bad cholesterol” for a long time and lipid-lowering drugs have been prescribed even at normal levels of serum cholesterol[6], the impact can be substantial. Therefore, the underlying relationship between LDL-C and all-cause mortality needs to be clarified in large prospective cohorts.

For peer review only - http://bmjopen.bmj.com/site/about/guidelines.xhtml

In this study, we aimed to investigate whether LDL-C levels are associated with all-cause mortality among middle-aged and elderly Chinese men and women, based on the longitudinal data from the China Health and Retirement Longitudinal Study (CHARLS).

Methods

Study design

As a nationally representative longitudinal study, CHARLS is designed to collect a wide range of information on the economic standing, physical and psychological health, demographics and social networks of a middle-aged and elderly Chinese population (aged ≥45years)[7]. The national baseline survey (wave 1) was conducted between June 2011 and March 2012 and included 17,708 respondents. The second wave (wave 2) was carried out in 2013-2014, the third wave (wave 3) in 2014-2015 and the fourth wave (wave 4) in 2015-2016. The detailed design of CHARLS can be referred to a previous publication[7]. This study was approved by Biomedical Ethics Review Committee of Peking University, and all participants signed informed consents.

Study population

All participants recruited in the national baseline survey were included if they met the following criteria: 1) aged ≥45years, 2) measured plasma levels of LDL-C in wave 1, 3) successfully followed up in at least one of the subsequent three waves, and 4) without lipid-lowering interventions. Finally, 10510 participants, including 4981 men and 5529 women, were included for subsequent analysis (Figure 1).

Plasma LDL-C measurements and other covariates

Plasma samples were collected by medically-trained staff and then stored at –80°C until assayed at Capital Medical University (CMU) laboratory. LDL-C was measured by the enzymatic colormetric test, with an analytical range of 3–400 mg/L and between-assay coefficient of variation of 1.20%. During the testing of the CHARLS study samples, quality control (QC) samples were used daily. All test results from QC samples were within two standard deviations of mean QC control

For peer review only - http://bmjopen.bmj.com/site/about/guidelines.xhtml

concentrations. The other covariates collected included age, gender, smoking status, drinking status, body mass index (BMI), educational level, household income, living alone status, rural residence, activity of daily living (ADL) disability, high-density lipoprotein cholesterol (HDL-C), triglyceride, hemoglobin, hypertension (defined by a history of hypertension, or systolic blood pressure (SBP) $\geq$ 140mmHg, or diastolic blood pressure (DBP) $\geq$ 90 mmHg), high blood sugar (HBS)/diabetes (defined by a history of HBS/diabetes, or fasting blood glucose $\geq$ 6.1mmol/L, or non-fasting blood glucose $\geq$ 7.8mmol/L), a history of cancer, cardiovascular disease, stroke, asthma, lung disease, liver disease, digestive disease, kidney disease, arthritis, memory problem and psychological problem. Activity of daily living (ADL) covers the following items: dressing, bathing and showering, eating, getting in/out bed, using the toilet and controlling urination or defecation. Every item in the ADL scale has a four-scale answer for each question: “no difficulty”, “have difficulty but can still do it”, “have difficulty and need help”, and “can not do it”. ADL was assigned a value of 0 if the respondents had no difficulty in all these activities and 1 otherwise. Hand grip strength was measured with a dynamometer (Yuejian™ WL-1000, Nantong, China) in kilograms (kg) twice on each hand. The mean score of two measures in the dominant hand was calculated to define hand grip strength in this study.

All-cause mortality follow-up

Participants enrolled in wave 1 were followed up in subsequent three waves. In wave 2, both the interview status (dead or alive) and death time were recorded. In waves 3 and 4, only the interview status was recorded. For those who had the exact time on all-cause death in wave 2, the survival time was defined as the interval between the interview time of wave 1 and the death time. If the exact death time was not available in waves 3 and 4, the survival time was computed as the median of the interval between wave 1 and the specific wave with death information. For those who did not die during the follow-up period, the survival time was defined as the interval between wave 1 and the last interview wave with follow-up information.

Patient and public involvement

Anonymised participant data were used in this study. Patients and the public

For peer review only - http://bmjopen.bmj.com/site/about/guidelines.xhtml

were not involved in the design or conduct, or reporting, or dissemination plans of the study.

Statistical analysis

Data were presented as median (P_{25}~P_{75}) for continuous variables and frequency (percentage) for categorical variables. Baseline characteristics between or among groups were compared by the Wilcoxon rank sum test or Kruskal-Wallis rank sum test for continuous variables and by the chi-square test for categorical variables. The Cox proportional hazard ratio model was used to estimate the hazard ratios (HRs) and 95% confidence intervals (CIs) of LDL-C quintiles. In addition, the association between all-cause mortality and LDL-C on a continuous scale was further examined using restricted cubic splines (RCS) incorporated in Cox proportional hazards models. Bayesian Information Criterion (BIC) was used to determine the optimal number of knots in RCS. In this study, 3 knots were used in all RCS analyses, with knot locations at the 10th, 50th, and 90th percentiles of LDL-C. To be consistent with quintile analyses, the reference point was the 20th percentile of LDL-C in both men and women. All statistical analyses were performed by SAS statistical software (version 9.4, Cary, NC). All P values were 2-tailed, and the significance level was set at 0.05.

Results

Baseline characteristics of the study population

A total of 4981 men and 5529 women were eligible for the final analysis. The median of LDL-C levels in women was significantly higher than that in men ($P < 0.0001$, Supplementary Figure S1). Compared with women, men were older, had smaller BMI values and possessed greater smoking rate and drinking rate (all $P < 0.0001$). The prevalence rates of heart disease, arthritis and digestive disease in women were higher than those in men, but the prevalence rates of asthma and lung disease were lower in women (all $P < 0.0001$, Table 1).

Characteristics of men and women according to the quintiles of LDL-C levels

After stratification by the quintiles of LDL-C levels, BMI, SBP, DBP and

For peer review only - http://bmjopen.bmj.com/site/about/guidelines.xhtml

hemoglobin in men were elevated with ascending quintiles as a whole (All P<0.001) (Table 2). The prevalence of HBS/diabetes was highest in the bottom quintile of LDL-C and lowest in the fourth quintile, with prevalence rates of 34.81% and 27.06% respectively. There were no statistical differences among LDL-C quintiles for many other characteristics (e.g., age, smoking, drinking, ADL disability, living alone, stroke, cancer, heart disease, lung disease, liver disease, kidney disease, digestive disease, asthma, arthritis, psychological problem and memory problem) (All P> 0.05).

In women, LDL-C quintiles were positively associated with age, BMI, SBP, DBP and hemoglobin (All P <0.001) (Table 3). The prevalence rates of HBS/diabetes and liver disease in women were significantly different among different LDL-C quintiles (All P<0.001). For the remaining variables (e.g., smoking, drinking, household income, ADL disability, educational level, rural residence, hand grip strength, stroke, cancer, heart disease, lung disease, kidney disease, digestive disease, asthma, arthritis, psychological problem and memory problem), no differences were observed (All P > 0.05).

Associations of LDL-C levels with all-cause mortality

In men, 305 out of 4981 participants died during a four-year follow-up. The mortality rates were declining with ascending quintiles (Table 4). Compared with the first quintile, the univariate HRs (95%CIs) were 0.733(0.533~1.007) for the second quintile; 0.639(0.458~0.892) for the third quintile; 0.519(0.364~0.739) for the fourth quintile and 0.512(0.359~0.732) for the fifth quintile. After adjustment for a series of potential confounders, the non-linear association between LDL-C and all-cause mortality was observed. As compared with the first quintile, the multivariable HRs (95%CIs) were as follows: second quintile, 0.818(0.531~1.260); third quintile, 0.782(0.507~1.208); fourth quintile, 0.605(0.381~0.962); fifth quintile, 0.803(0.506~1.274). In women, there were 219 deaths during a four-year follow-up. The mortality rate was highest in the first quintile. After adjustment for potential confounders, no quintile showed significant lower mortality rates compared with the first quintile (all P> 0.05) (Table 4).

The quintile analysis indicated that the relationship between LDL-C with

For peer review only - http://bmjopen.bmj.com/site/about/guidelines.xhtml

all-cause mortality might be non-linear. Therefore, RCS was further performed to investigate the association between all-cause mortality and LDL-C on a continuous scale. The results from RCS showed that when the 20th percentile of LDL-C levels (84mg/dL) was used as the reference, lower LDL-C (<84mg/dL) was associated with higher risk of 4-year all-cause mortality in men, and moderately higher LDL-C (84-135mg/dL) possessed lower total mortality risk, but the association was not statistically significant for much higher LDL-C concentrations (>135mg/dL) (Figure 2). The sub-group analyses by age indicated that when the 20th percentile of LDL-C levels was taken as the reference, a lower level of LDL-C was associated with a higher risk of 4-year all-cause mortality in both middle-aged (45~60 years) and elderly (≥60 years) men (Figure 3). For women, LDL-C was not significantly associated with 4-year all-cause mortality (Figures 2 and 3).

In addition, we found that 125 out of 4981 men and 89 out of 5529 women had LDL-C < 50mg/dL. When participants with LDL-C < 50 mg/dL were excluded, the hazard ratio of the fourth LDL-C quintile in men was changed a little with marginal statistical significance (*P*=0.0698, Supplementary Table S1). Moreover, no interactions were found between LDL-C and potential risk factors of mortality, with the exception that the interaction between LDL-C and smoking in women was statistical significant (*P*=0.0498, Supplementary Table S2).

Discussion

In this study, we investigated the relationship between LDL-C and 4-year all-cause mortality among the middle-aged and elderly Chinese population. In men, a very low level of LDL-C was associated with increased mortality risk. In women, LDL-C was not significantly associated with 4-year all-cause mortality.

Low-density lipoprotein has been well established as an important cause of cardiovascular disease (CVD) for decades[1]. Since CVD is the leading cause of mortality throughout the world, it is logically reasonable that increased LDL-C should contribute to increased CVD mortality and possibly all-cause mortality. Indeed, evidences from prospective epidemiologic studies showed a positive association

For peer review only - http://bmjopen.bmj.com/site/about/guidelines.xhtml

between non-HDL-C concentration and ischaemic heart disease mortality[8]. However, non-HDL-C includes both LDL-C and very low-density lipoprotein cholesterol (VLDL-C). It is surprising that the direct association of LDL-C with CVD mortality was not consistently reported among studies. Abdullah *et al.* (2018) demonstrated that LDL-C was independently associated with CVD mortality in a low 10-year risk cohort with long-term follow-up[9]. By contrast, Tikhonof *et al.* (2005) reported that the elderly subjects (≥ 65 years) possessed the highest CVD mortality in the lowest LDL-C quartile[10]. Meanwhile, many other studies also found no association between LDL-C and CVD mortality[11-13]. When the results about the association of LDL-C with CVD mortality were inconsistent, it is more surprising to find that few studies have reported the positive association between LDL-C and all-cause mortality. In the study by Abdullah *et al.* (2018), there were already no associations or minimal positive associations between high LDL-C categories and all-cause mortality even in univariable Cox analyses[9]. Most remarkably, results of multivariable Cox analyses in this study were not provided for all-cause mortality, which could exert a substantial impact on the final association[14]. Actually, a large number of studies reported no association or even an inverse association between LDL-C and all-cause mortality, which has been summarized in a systematic review by Ravnskov *et al.* (2016)[5]. Therefore, very low LDL-C in populations not on lipid therapy may be associated with harm.

It should be noted that the confounding effect of statin treatment should be minimized, as this study excluded those who used lipid-lowering interventions. There was also no association between baseline LDL-C and the presence of cancer, stroke and heart disease in men and women (Tables 2 and 3). Moreover, when participants who had died during the first observation year were excluded, this relationship was not changed (Supplementary Table S3 and Figure S2). This could relieve the concern that serious diseases may lower cholesterol soon before death occurs. One of the possible reasons for the difference between men and women may be due to fewer death events in women than in men, which might result in insufficient power for the association.

For peer review only - http://bmjopen.bmj.com/site/about/guidelines.xhtml

Several explanations for the unfavorable effects of low LDL-C levels may be proposed. LDL-C has been suggested to play an important role in host defense against both bacterial and viral pathogens[15]. Indeed, many animal and laboratory experiments have shown that LDL could bind to and inactivate a broad range of microorganisms and their toxic products[16-18]. This hypothesis may be further supported by the recent finding that LDL-C was associated with reduced infectious mortality based on the data from 37,250 patients in the international Monitoring Dialysis Outcomes (MONDO) database[2]. In addition, it has been proposed that LDL-C may have the potential to protect against cancer as many cancer types are caused by viruses[19]. Ravnskov *et al.* (2012) reviewed nine cohort studies including more than 140,000 individuals followed for 10–30 years and found that low cholesterol was associated with cancer[20]. Moreover, cholesterol-lowering experiments on rodents have led to cancer as well[21]. In agreement with these findings, individuals with familial hypercholesterolaemia have been found to possess significantly lower cancer mortality[22]. Therefore, lower LDL-C may contribute to a higher risk of death from infection and cancer, which in turn results in increased all-cause mortality.

This study demonstrated that middle-aged and elderly Chinese men with very low LDL-C had an increased risk of all-cause mortality, which calls for special attention to be paid to the possible harmful effect of a very low level of LDL-C. However, some limitations should be noted. First, the follow-up period was limited to 4 years. For a longer follow-up time, the associations between LDL-C and all-cause mortality in women might be displayed. Second, cause-specific mortality data were not available for the time being, preventing the analysis of the association between LDL-C and cause-specific mortality. Third, there are issues of multiple testing for comparisons of characteristics among LDL-C quintiles, which could result in Type I error inflation. Fourth, some of the measured co-morbidities were not specified and detailed in the database, such as lung disease, digestive disease, liver disease, kidney disease, psychological problem and memory problem. At last, well-designed, large-scale population studies are needed to formulate the specific LDL-C level(s)

For peer review only - http://bmjopen.bmj.com/site/about/guidelines.xhtml

threshold for mortality risk in the future.

In China, compared with moderately elevated LDL-C (e.g., 117-137mg/dL), a lower plasma level of LDL-C (e.g., ≤84mg/dL) was associated with an increased risk of 4-year all-cause mortality in middle-aged and elderly men. The findings in this study may suggest the potential harmful effect of a quite low level of LDL-C. More prospective and well-designed studies are needed to validate the relationship between LDL-C and mortality.

Acknowledgements

This analysis uses data or information from the Harmonized CHARLS dataset and Codebook, Version C as of April 2018 developed by the Gateway to Global Aging Data. The development of the Harmonized CHARLS was funded by the National Institute on Ageing (R01 AG030153, RC2 AG036619, R03 AG043052). For more information, please refer to www.g2aging.org.

Funding

This work was supported by National Natural Science Foundation of China (81703316 to C.K., 81703322 to Y.W.) and Natural Science Foundation of Jiangsu Province (BK20170350).

Competing interests

None declared.

Patient consent for publication

Obtained

Contributors

CK and YS conceived and designed the research; LZ and CK wrote the manuscript; and YW and SY performed the data analysis. All authors contributed to the interpretations of the findings. All authors reviewed the manuscript.

For peer review only - http://bmjopen.bmj.com/site/about/guidelines.xhtml

Ethics approval

CHARLS was approved by Biomedical Ethics Review Committee of Peking University, and all participants signed informed consents.

Data sharing statement

The data used and analysed in this study are publicly available from the China Health and Retirement Longitudinal Study (http://charls.pku.edu.cn/zh-CN).

Figure legends

Figure 1 Flowchart on the selection of eligible participants.

Figure 2 Results from restricted cubic splines for the association between LDL-C and 4-year all-cause mortality in men and women, respectively. The multivariable models were adjusted for age, smoking, drinking, BMI, marital status, household income, educational level, rural residence, ADL disability, hand grip strength, HDL-C, triglyceride, hemoglobin, hypertension, HBS/diabetes, history of stroke, cancer, heart disease, lung disease, liver disease, kidney disease, digestive disease, asthma, arthritis, psychological problem and memory problem.

Figure 3 Results from restricted cubic spline for the association between LDL-C and 4-year all-cause mortality for middle-aged (45~60 years old) and elderly (≥ 60 years old) people, respectively. The multivariable models were adjusted for age, smoking, drinking, BMI, marital status, household income, educational level, rural residence, ADL disability, hand grip strength, HDL-C, triglyceride, hemoglobin, hypertension, HBS/diabetes, history of stroke, cancer, heart disease, lung disease, liver disease, kidney disease, digestive disease, asthma, arthritis, psychological problem and memory problem.

References

1. Ference BA, Ginsberg HN, Graham I, et al. Low-density lipoproteins cause atherosclerotic cardiovascular disease. 1. Evidence from genetic, epidemiologic, and clinical studies. A consensus statement from the European Atherosclerosis Society Consensus Panel. Eur Heart J

For peer review only - http://bmjopen.bmj.com/site/about/guidelines.xhtml

2017;**38**(32):2459-72 |.

2. Kaysen GA, Ye X, Raimann JG, et al. Lipid levels are inversely associated with infectious and all-cause mortality: international MONDO study results. Journal of lipid research 2018;**59**(8):1519-28|.
3. Chang JJ, Katsanos AH, Khorchid Y, et al. Higher low-density lipoprotein cholesterol levels are associated with decreased mortality in patients with intracerebral hemorrhage. Atherosclerosis 2018;**269**:14-20|.
4. Charach G, George J, Roth A, et al. Baseline low-density lipoprotein cholesterol levels and outcome in patients with heart failure. The American journal of cardiology 2010;**105**(1):100-4|.
5. Ravnskov U, Diamond DM, Hama R, et al. Lack of an association or an inverse association between low-density-lipoprotein cholesterol and mortality in the elderly: a systematic review. Bmj Open 2016;**6**(6): e010401.
6. Heart Protection Study Collaborative G. MRC/BHF Heart Protection Study of cholesterol lowering with simvastatin in 20,536 high-risk individuals: a randomised placebo-controlled trial. Lancet 2002;**360**(9326):7-22|.
7. Zhao Y, Hu Y, Smith JP, et al. Cohort profile: the China Health and Retirement Longitudinal Study (CHARLS). Int J Epidemiol 2014;**43**(1):61-8|.
8. Prospective Studies C, Lewington S, Whitlock G, et al. Blood cholesterol and vascular mortality by age, sex, and blood pressure: a meta-analysis of individual data from 61 prospective studies with 55,000 vascular deaths. Lancet 2007;**370**(9602):1829-39|.
9. Abdullah SM, Defina LF, Leonard D, et al. Long-Term Association of Low-Density Lipoprotein Cholesterol With Cardiovascular Mortality in Individuals at Low 10-Year Risk of Atherosclerotic Cardiovascular Disease. Circulation 2018;**138**(21):2315-25|.
10. Tikhonoff V, Casiglia E, Mazza A, et al. Low-density lipoprotein cholesterol and mortality in older people. Journal of the American Geriatrics Society 2005;**53**(12):2159-64|.
11. Raiha I, Marniemi J, Puukka P, et al. Effect of serum lipids, lipoproteins, and apolipoproteins on vascular and nonvascular mortality in the elderly. Arteriosclerosis, thrombosis, and vascular biology 1997;**17**(7):1224-32.
12. Upmeier E, Lavonius S, Lehtonen A, et al. Serum lipids and their association with mortality in the elderly: a prospective cohort study. Aging Clin Exp Res 2009;**21**(6):424-30|.
13. Blekkenhorst LC, Prince RL, Hodgson JM, et al. Dietary saturated fat intake and atherosclerotic vascular disease mortality in elderly women: a prospective cohort study. The American journal of clinical nutrition 2015;**101**(6):1263-8|.
14. Ke CF, Shen YP. Letter by Ke and Shen Regarding Article, "Long-Term Association of Low-Density Lipoprotein Cholesterol With Cardiovascular Mortality in Individuals at Low 10-Year Risk of Atherosclerotic Cardiovascular Disease: Results From the Cooper Center Longitudinal Study". Circulation 2019;**139**(18):2190-91|.
15. Feingold KR, Grunfeld C. Lipids: a key player in the battle between the host and microorganisms. Journal of lipid research 2012;**53**(12):2487-89|.
16. Han RL. Plasma lipoproteins are important components of the immune system. Microbiol Immunol 2010;**54**(4):246-53|.
17. Khovidhunkit W, Kim MS, Memon RA, et al. Effects of infection and inflammation on lipid and lipoprotein metabolism: mechanisms and consequences to the host. Journal of lipid research 2004;**45**(7):1169-96|.

For peer review only - http://bmjopen.bmj.com/site/about/guidelines.xhtml

For peer review only

18. Ravnskov U, McCully KS. Review and Hypothesis: Vulnerable plaque formation from obstruction of Vasa vasorum by homocysteinylated and oxidized lipoprotein aggregates complexed with microbial remnants and LDL autoantibodies. Annals of clinical and laboratory science 2009;**39**(1):3-16.

19. Read SA, Douglas MW. Virus induced inflammation and cancer development. Cancer letters 2014;**345**(2):174-81|.

20. Ravnskov U, McCully KS, Rosch PJ. The statin-low cholesterol-cancer conundrum. QJM : monthly journal of the Association of Physicians 2012;**105**(4):383-8.

21. Newman TB, Hulley SB. Carcinogenicity of lipid-lowering drugs. Jama 1996;**275**(1):55-60.

22. Neil HA, Hawkins MM, Durrington PN, et al. Non-coronary heart disease mortality and risk of fatal cancer in patients with treated heterozygous familial hypercholesterolaemia: a prospective registry study. Atherosclerosis 2005;**179**(2):293-7.

For peer review only

For peer review only - http://bmjopen.bmj.com/site/about/guidelines.xhtml

Table 1 Characteristics of the study population

Characteristics	Men (*n*=4981)	Women (*n*=5529)	*P* value
Age-yr	59 (53~66)	57 (51~65)	<0.0001
BMI-kg/m^2	22.40 (20.35~24.83)	23.51 (21.17~26.14)	<0.0001
SBP-mmHg	127.67 (115.67~141.67)	127.00 (114.00~143.33)	0.3764
DBP-mmHg	75.33 (67.67~83.67)	74.33 (67.00~82.67)	0.0017
Hand grip strength (kg)	36.85 (30.50~43.00)	25.00 (20.00~29.50)	<0.0001
Above-average household income-no. (%)	2104 (49.68)	2360 (50.32)	0.5468
Education level-no. (%)			<0.0001
1	4287 (86.07)	5134 (92.86)	
2	592 (11.89)	347 (6.28)	
3	102 (2.05)	48 (0.87)	
ADL disability-no. (%)	716 (14.55)	1011 (18.57)	<0.0001
Living alone-no. (%)	471 (9.46)	832 (15.05)	<0.0001
Rural residence-no. (%)	3282 (65.89)	3524 (63.74)	0.0210
Lifestyle-no. (%)			
Smoking ever	3738 (75.24)	420 (7.63)	<0.0001
Drinking ever	3297 (66.43)	826 (15.01)	<0.0001
Disease history-no. (%)			
Hypertension	1904 (43.35)	2245 (45.34)	0.0530
HBS/Diabetes	1460 (30.16)	1564 (29.19)	0.2846
Cancer	40 (0.81)	67 (1.22)	0.0372
Stroke	133 (2.69)	136 (2.48)	0.4958
Heart disease	499 (10.10)	738 (13.47)	<0.0001
Lung disease	620 (12.54)	508 (9.26)	<0.0001
Arthritis	1548 (31.23)	2243 (40.80)	<0.0001
Liver disease	197 (4.00)	188 (3.44)	0.1326
Kidney disease	309 (6.27)	315 (5.75)	0.2701
Digestive disease	1030 (20.79)	1393 (25.37)	<0.0001
Asthma	283 (5.72)	216 (3.93)	<0.0001
Psychological problem	60 (1.21)	94 (1.72)	0.0336
Memory problem	97 (1.96)	86 (1.57)	0.1238
Laboratory measurements			
LDL cholesterol-mg/dL	109.41 (88.92~131.06)	117.91 (96.26~141.50)	<0.0001
Triglyceride-mg/dL	96.46 (69.03~145.14)	110.63 (79.65~159.30)	<0.0001
HDL cholesterol-mg/dL	48.71 (39.43~59.54)	50.64 (41.75~60.31)	<0.0001
Hemoglobin-(g/dL)	15.10 (14.00~16.20)	13.60 (12.50~14.60)	<0.0001

ADL, activity of daily living; BMI, body mass index; SBP, systolic blood pressure; DBP, diastolic blood pressure; HBS, high blood sugar.

Educational level: 1, Less than lower secondary education; 2, Upper secondary & vocational training; 3, Tertiary education.

For peer review only

For peer review only - http://bmjopen.bmj.com/site/about/guidelines.xhtml

Table 2 Baseline characteristics of participants by quintiles of LDL-C in men

Characteristics	Quintile 1 (*n*=991) (≤83.89mg/dL)	Quintile 2 (*n*=1008) (83.89~101.68)	Quintile 3 (*n*=991) (101.68~117.14)	Quintile 4 (*n*=1004) (117.14~136.86)	Quintile 5 (*n*=987) (>136.86)	*P* value
Age-yr	59 (52~66)	59 (53~67)	59 (52~66)	59 (53~67)	59 (53~65)	0.5405
BMI-kg/m^2	21.57 (19.78~24.21)	22.18 (20.31~24.40)	22.36 (20.44~24.61)	22.64 (20.65~25.07)	23.27 (20.95~25.68)	<0.0001
SBP-mmHg	126.00 (114.00~140.00)	126.00 (115.33~139.67)	127.00 (114.67~142.33)	128.00 (115.67~142.50)	130.67 (118.00~144.00)	0.0002
DBP-mmHg	75.00 (66.67~83.33)	74.33 (66.67~82.00)	74.67 (67.33~83.33)	75.33 (67.67~83.67)	76.67 (69.33~85.33)	0.0002
Hand grip strength (kg)	35.93 (30.00~42.50)	36.00 (30.00~42.60)	36.93 (30.00~43.00)	37.50 (31.50~43.75)	37.50 (31.50~42.90)	0.0132
Above-average household income-no. (%)	402 (48.43)	413 (47.91)	407 (48.05)	430 (49.20)	452 (54.99)	0.0186
Educational level-no. (%)						0.0035
1	870 (87.79)	891 (88.39)	821 (82.85)	862 (85.86)	843 (85.41)	
2	105 (10.60)	94 (9.33)	145 (14.63)	123 (12.25)	125 (12.66)	
3	16 (1.61)	23 (2.28)	25 (2.52)	19 (1.93)	19 (1.93)	
ADL disability-no. (%)	149 (15.27)	163 (16.30)	148 (15.10)	141 (14.23)	115 (11.81)	0.0590
Living alone-no. (%)	101 (10.19)	112 (11.11)	85 (8.58)	94 (9.36)	79 (8.00)	0.1264
Rural residence-no. (%)	659 (66.50)	702 (69.64)	634 (63.98)	664 (66.14)	623 (63.12)	0.0216
Lifestyle-no. (%)						
Smoking ever	755 (76.57)	754 (75.02)	751 (75.94)	759 (75.75)	719 (72.92)	0.3788
Drinking ever	654 (66.40)	663 (66.10)	664 (67.14)	664 (66.40)	652 (66.13)	0.9890
Disease history-no. (%)						
Hypertension	359 (42.14)	349 (39.48)	385 (42.87)	397 (44.51)	414 (47.81)	0.0092
HBS/Diabetes	337 (34.81)	277 (28.35)	266 (27.85)	266 (27.06)	314 (32.78)	0.0003
Cancer	13 (1.32)	7 (0.70)	5 (0.51)	6 (0.60)	9 (0.92)	0.2728
Stroke	26 (2.64)	33 (3.31)	21 (2.13)	25 (2.50)	28 (2.85)	0.5818
Heart disease	86 (8.78)	98 (9.81)	103 (10.47)	113 (11.31)	99 (10.10)	0.4453
Lung disease	131 (13.37)	135 (13.46)	106 (10.77)	120 (12.00)	128 (13.09)	0.3170
Arthritis	300 (30.43)	332 (33.10)	280 (28.34)	312 (31.23)	324 (33.03)	0.1233
Liver disease	51 (5.23)	42 (4.19)	35 (3.57)	38 (3.81)	31 (3.18)	0.1855
Kidney disease	56 (5.73)	73 (7.29)	59 (6.02)	57 (5.71)	64 (6.56)	0.5518
Digestive disease	211 (21.46)	213 (21.24)	225 (22.82)	203 (20.28)	178 (18.14)	0.1264
Asthma	66 (6.76)	49 (4.89)	43 (4.36)	59 (5.91)	66 (6.72)	0.0754
Psychological problem	14 (1.43)	10 (1.00)	14 (1.42)	14 (1.40)	8 (0.82)	0.6092
Memory problem	25 (2.55)	13 (1.30)	21 (2.13)	19 (1.90)	19 (1.94)	0.3810
Laboratory measurements						
Triglyceride-mg/dL	96.46 (65.49~177.88)	88.50 (64.61~129.21)	92.93 (69.03~138.95)	97.35 (72.57~136.29)	108.86 (79.65~ 152.22)	<0.0001
HDL cholesterol-mg/dL	47.55	49.48	47.93	49.10	48.71	0.0061

For peer review only - http://bmjopen.bmj.com/site/about/guidelines.xhtml

	(35.95~59.92)	(39.43~60.70)	(39.43~58.76)	(39.82~59.54)	(41.37~58.76)	
Hemoglobin-(g/dL)	14.80 (13.60~16.00)	14.90 (13.80~16.10)	15.00 (14.00~16.20)	15.10 (14.00~16.30)	15.40 (14.30~16.50)	<0.0001
LDL cholesterol-mg/dL	71.91 (61.47~78.09)	93.56 (88.92~97.81)	109.41 (105.54~113.27)	126.42 (121.39~131.44)	153.87 (144.20~170.49)	<0.0001

Table 3 Baseline characteristics of participants by quintiles of LDL-C in women

Characteristics	Quintile 1 (*n*=1114) (≤91.24mg/dL)	Quintile 2 (*n*=1102) (91.24~109.41)	Quintile 3 (*n*=1096) (109.41~126.03)	Quintile 4 (*n*=1111) (126.03~147.68)	Quintile 5 (*n*=1106) (>147.68)	*P* value
Age-yr	56 (49~64)	56 (49~64)	57 (50~63)	58 (52~65)	59 (53~66)	<0.0001
BMI-kg/m^2	23.15 (20.90~25.76)	23.30 (21.01~25.82)	23.39 (21.10~26.02)	23.63 (21.32~26.26)	24.12 (21.57~26.83)	<0.0001
SBP-mmHg	124.67 (112.00~141.00)	125.67 (113.00~142.33)	127.00 (114.67~143.00)	127.67 (114.67~142.33)	130.00 (117.00~146.00)	<0.0001
DBP-mmHg	73.33 (65.67~81.67)	74.33 (66.67~82.67)	74.00 (67.33~82.67)	74.67 (67.67~82.67)	75.33 (67.67~83.33)	0.0103
Hand grip strength (kg)	24.75 (19.80~30.00)	25.00 (20.00~29.50)	25.50 (20.55~30.00)	24.95 (20.00~29.50)	24.50 (20.25~29.50)	0.3187
Above-average household income-no. (%)	458 (48.21)	482 (50.42)	461 (48.99)	497 (53.61)	462 (50.44)	0.1720
Education level-no. (%)						0.4079
1	1023 (91.83)	1030 (93.47)	1023 (93.34)	1025 (92.26)	1033 (93.40)	
2	76 (6.82)	67 (6.08)	63 (5.75)	76 (6.84)	65 (5.88)	
3	15 (1.35)	5 (0.45)	10 (0.91)	10 (0.90)	8 (0.72)	
ADL disability-no. (%)	216 (19.69)	183 (16.90)	188 (17.59)	215 (19.58)	209 (19.05)	0.3416
Living alone-no. (%)	155 (13.91)	162 (14.70)	143 (13.05)	186 (16.74)	186 (16.82)	0.0429
Rural residence-no. (%)	715 (64.18)	695 (63.07)	726 (66.24)	706 (63.55)	682 (61.66)	0.2526
Lifestyle-no. (%)						
Smoking ever	71 (6.39)	93 (8.49)	78 (7.15)	79 (7.14)	99 (8.97)	0.1294
Drinking ever	173 (15.61)	160 (14.61)	169 (15.50)	165 (14.91)	159 (14.40)	0.9112
Disease history-no. (%)						
Hypertension	414 (41.69)	440 (44.58)	431 (44.21)	451 (45.46)	509 (50.70)	0.0014
HBS/Diabetes	310 (28.78)	290 (27.46)	280 (26.19)	311 (28.88)	373 (34.53)	0.0003
Cancer	14 (1.27)	15 (1.38)	18 (1.66)	14 (1.27)	6 (0.54)	0.1876
Stroke	31 (2.80)	16 (1.47)	27 (2.47)	31 (2.81)	31 (2.81)	0.1906
Heart disease	144 (13.08)	135 (12.41)	140 (12.89)	166 (15.09)	153 (13.87)	0.3873
Lung disease	110 (9.94)	112 (10.29)	103 (9.45)	91 (8.27)	92 (8.36)	0.3566
Arthritis	454 (41.05)	450 (41.21)	421 (38.55)	454 (41.05)	464 (42.14)	0.5189
Liver disease	53 (4.80)	40 (3.69)	25 (2.31)	43 (3.91)	27 (2.46)	0.0060
Kidney disease	74 (6.71)	62 (5.69)	57 (5.24)	66 (6.02)	56 (5.09)	0.4886

For peer review only

Digestive disease	279 (25.20)	267 (24.45)	269 (24.70)	299 (27.13)	279 (25.34)	0.6323
Asthma	43 (3.89)	43 (3.94)	44 (4.03)	39 (3.54)	47 (4.26)	0.9384
Psychological problem	16 (1.45)	19 (1.74)	23 (2.12)	17 (1.54)	19 (1.73)	0.7943
Memory problem	18 (1.63)	19 (1.74)	11 (1.01)	15 (1.36)	23 (2.09)	0.3245
Laboratory measurements						
Triglyceride-mg/dL	110.63 (73.46~192.93)	103.54 (74.34~153.99)	107.97 (77.88~152.22)	110.63 (82.31~151.34)	125.23 (92.93~162.84)	<0.0001
HDL cholesterol-mg/dL	46.39 (35.57~57.60)	50.64 (40.98~60.70)	51.42 (42.53~60.70)	51.80 (43.69~61.08)	51.80 (44.46~60.31)	<0.0001
Hemoglobin-(g/dL)	13.20 (12.10~14.30)	13.30 (12.30~14.50)	13.60 (12.60~14.70)	13.70 (12.80~14.60)	13.80 (12.80~14.80)	<0.0001
LDL cholesterol-mg/dL	78.48 (68.04~85.83)	100.90 (96.26~105.54)	117.91 (113.66~121.78)	135.70 (130.28~141.50)	165.08 (155.03~179.00)	<0.0001

Table 4 Associations between all-cause mortality and LDL-C

	Total	Deaths (%)	Unadjusted HR (95%CI)	Unadjusted *P* value	Adjusted* HR (95%CI)	Adjusted* *P* value
Men						
Q1	991	88(8.88)	1	-	1	-
Q2	1008	67(6.65)	0.733(0.533~1.007)	0.0554	0.818(0.531~1.260)	0.3619
Q3	991	57(5.75)	0.639(0.458~0.892)	0.0084	0.782(0.507~1.208)	0.2677
Q4	1004	47(4.68)	0.519(0.364~0.739)	0.0003	0.605(0.381~0.962)	0.0335
Q5	987	46(4.66)	0.512(0.359~0.732)	0.0002	0.803(0.506~1.274)	0.3520
Women						
Q1	1114	52(4.67)	1	-	1	-
Q2	1102	49(4.45)	0.960(0.650~1.419)	0.8394	1.245(0.749~2.071)	0.3985
Q3	1096	29(2.65)	0.565(0.359~0.890)	0.0138	0.626(0.345~1.136)	0.1233
Q4	1111	41(3.69)	0.792(0.526~1.192)	0.2632	0.852(0.489~1.483)	0.5704
Q5	1106	48(4.34)	0.926(0.625~1.371)	0.7007	0.958(0.563~1.630)	0.8736

*Adjusted for age, smoking, drinking, BMI, marital status, household income, educational level, rural residence, ADL disability, hand grip strength, HDL-C, triglyceride, hemoglobin, hypertension, HBS/diabetes, history of stroke, cancer, heart disease, lung disease, liver disease, kidney disease, digestive disease, asthma, arthritis, psychological problem and memory problem.

For peer review only - http://bmjopen.bmj.com/site/about/guidelines.xhtml

For peer review only

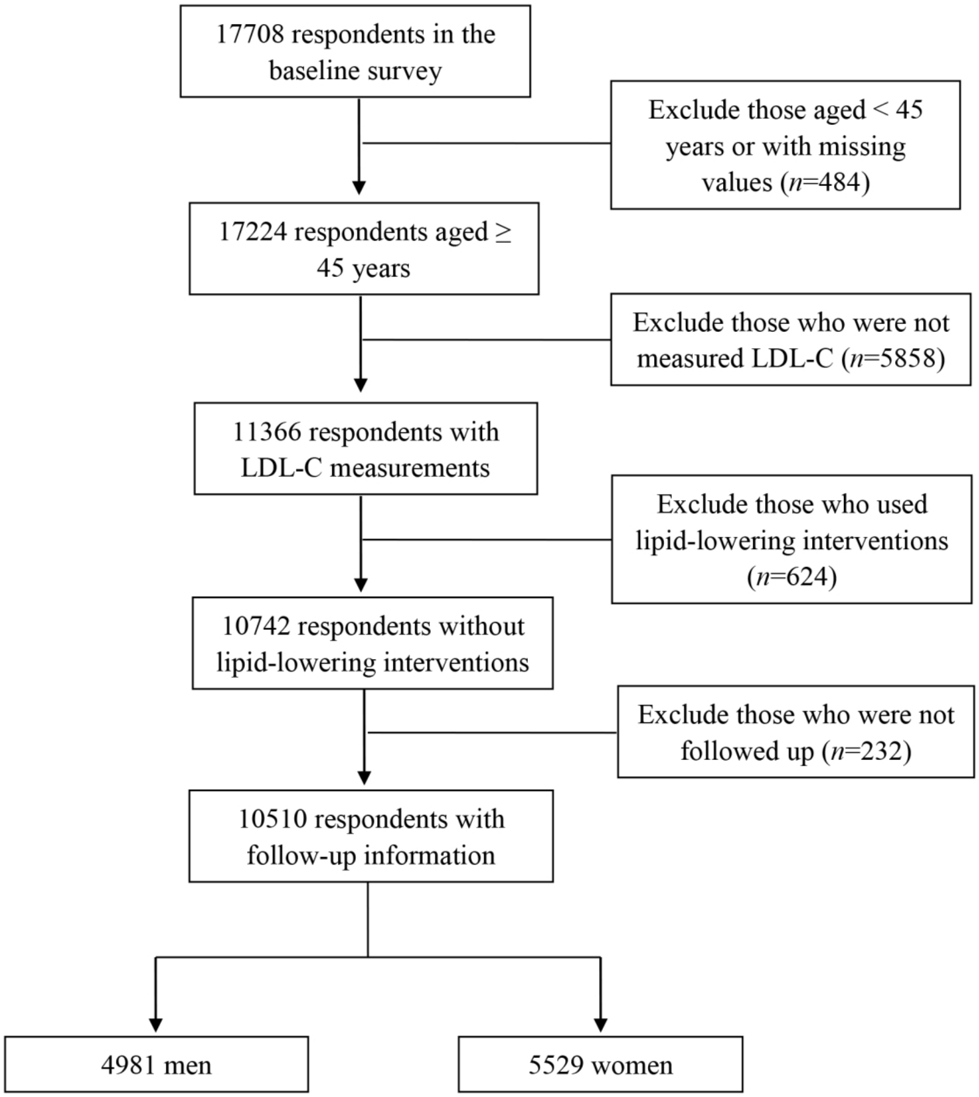

Figure 1 Flowchart on the selection of eligible participants.

151x168mm (300 x 300 DPI)

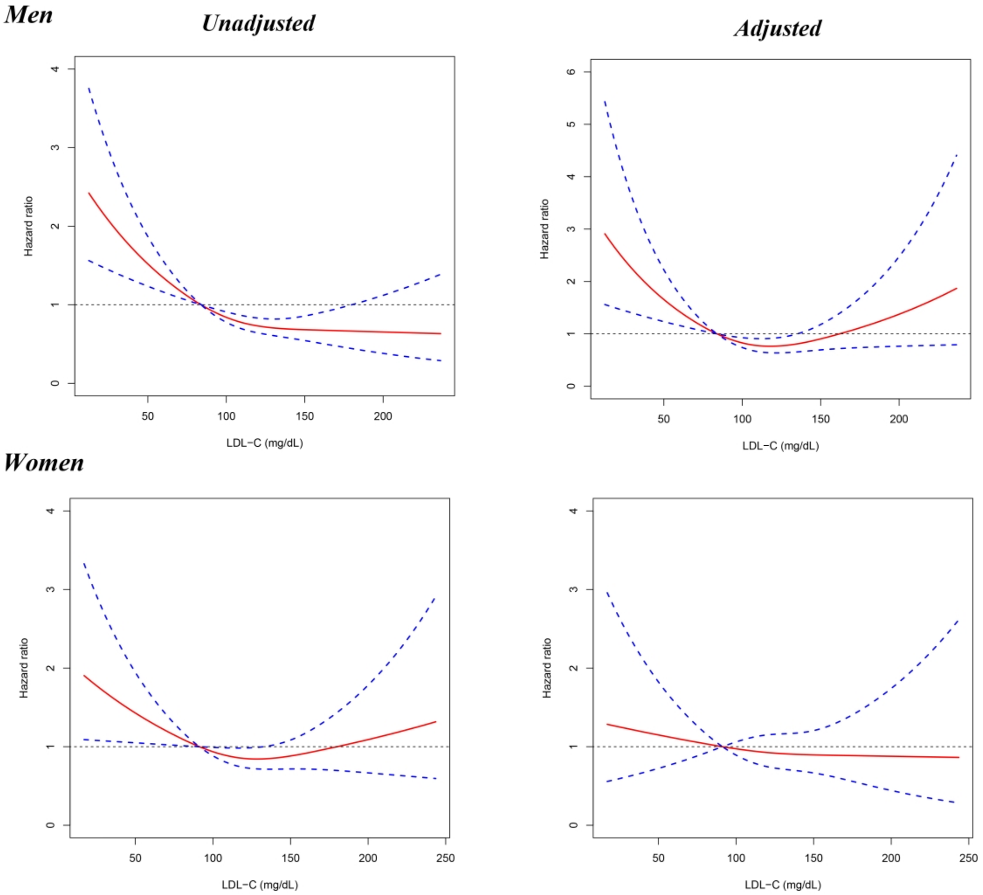

Figure 2 Results from restricted cubic splines for the association between LDL-C and 4-year all-cause mortality in men and women, respectively. The multivariable models were adjusted for age, smoking, drinking, BMI, marital status, household income, educational level, rural residence, ADL disability, hand grip strength, HDL-C, triglyceride, hemoglobin, hypertension, HBS/diabetes, history of stroke, cancer, heart disease, lung disease, liver disease, kidney disease, digestive disease, asthma, arthritis, psychological problem and memory problem.

381x355mm (300 x 300 DPI)

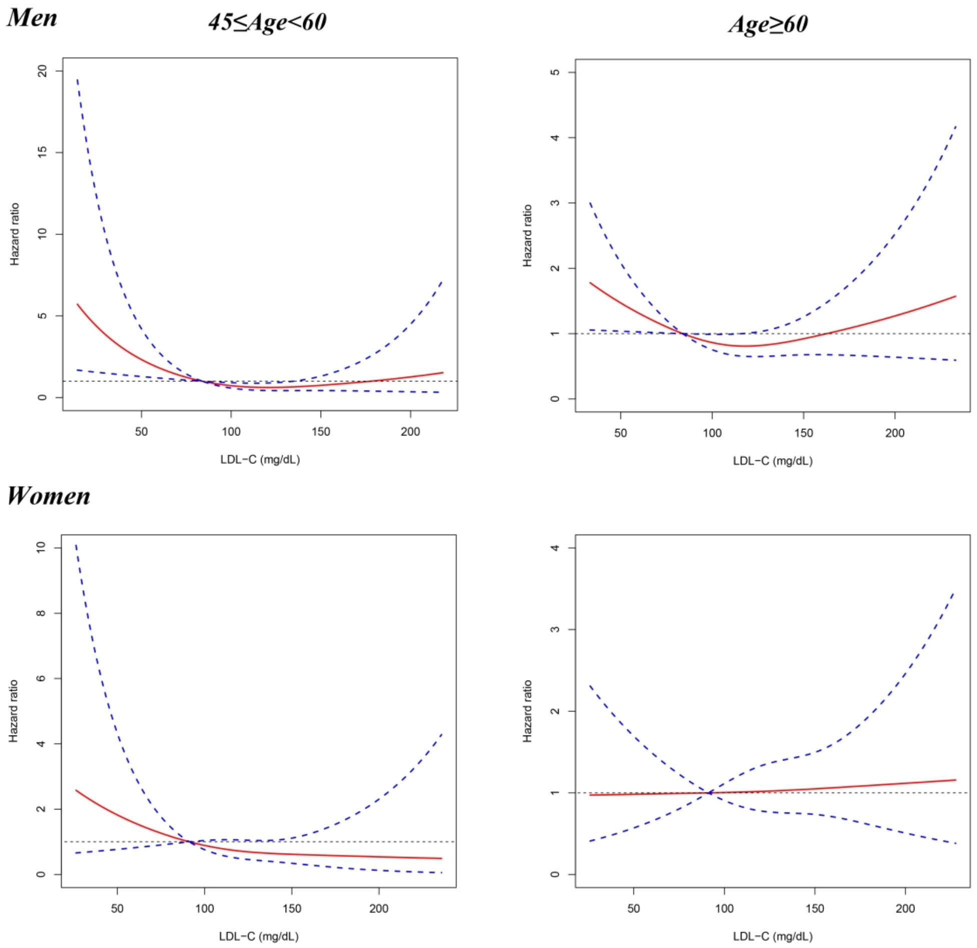

Figure 3 Results from restricted cubic spline for the association between LDL-C and 4-year all-cause mortality for middle-aged (45~60 years old) and elderly (≥ 60 years old) people, respectively. The multivariable models were adjusted for age, smoking, drinking, BMI, marital status, household income, educational level, rural residence, ADL disability, hand grip strength, HDL-C, triglyceride, hemoglobin, hypertension, HBS/diabetes, history of stroke, cancer, heart disease, lung disease, liver disease, kidney disease, digestive disease, asthma, arthritis, psychological problem and memory problem.

366x355mm (300 x 300 DPI)

Supplementary materials

Low-density lipoprotein cholesterol and all-cause mortality: findings from the China Health and Retirement Longitudinal Study

Liang Zhou [1], Ying Wu [2], Shaobo Yu [3], Yueping Shen [4] and Chaofu Ke [4]

[1] Liyang Center for Disease Control and Prevention, 55 Nanhuan Road, Liyang 213371, P. R. China.

[2] State Key Laboratory of Organ Failure Research, Department of Biostatistics, Guangdong Provincial Key Laboratory of Tropical Disease Research, School of Public Health, Southern Medical University, Guangzhou 510515, China.

[3] Medical College of Soochow University, Suzhou 215123, P. R. China.

[4] Department of Epidemiology and Biostatistics, School of Public Health, Medical College of Soochow University, 199 Renai Road, Suzhou 215123, P. R. China.

For peer review only

Supplementary Table S1 Associations between all-cause mortality and LDL-C#

	Total	Deaths (%)	Unadjusted		Adjusted*	
			HR (95%CI)	*P* value	HR (95%CI)	*P* value
Men						
Q1	866	75 (8.66)	1	-	1	-
Q2	1008	67(6.65)	0.750(0.539~1.042)	0.0865	0.863(0.551~1.351)	0.5180
Q3	991	57(5.75)	0.653(0.463~0.922)	0.0155	0.833(0.531~1.307)	0.4261
Q4	1004	47(4.68)	0.531(0.368~0.764)	0.0007	0.643(0.399~1.036)	0.0698
Q5	987	46(4.66)	0.524(0.363~0.756)	0.0006	0.861(0.533~1.390)	0.5406
Women						
Q1	1025	46 (4.49)	1	-	1	-
Q2	1102	49(4.45)	0.998(0.667~1.492)	0.9920	1.231(0.735~2.062)	0.4290
Q3	1096	29(2.65)	0.587(0.369~0.935)	0.0248	0.619(0.340~1.127)	0.1167
Q4	1111	41(3.69)	0.822(0.540~1.253)	0.3628	0.840(0.480~1.472)	0.5430
Q5	1106	48(4.34)	0.962(0.642~1.441)	0.8511	0.939(0.548~1.609)	0.8180

#Participants with LDL-C < 50 mg/dL were excluded.

*Adjusted for age, smoking, drinking, BMI, living alone, household income, educational level, rural residence, ADL disability, hand grip strength, HDL-C, triglyceride, hemoglobin, hypertension, HBS/diabetes, history of stroke, cancer, heart disease, lung disease, liver disease, kidney disease, digestive disease, asthma, arthritis, psychological problem and memory problem.

For peer review only

Supplementary Table S2 Analyses of interactions between LDL-C and potential risk factors

ID	Interaction term	*P* value in men	*P* value in women
1	LDL-C*age	*0.7323*	*0.0931*
2	LDL-C*obesity (BMI⩾28)	*0.4033*	*0.4825*
3	LDL-C*rural residence	*0.1426*	*0.8102*
4	LDL-C*ADL disability	*0.4108*	*0.5052*
5	LDL-C*smoking	*0.6150*	*0.0498*
6	LDL-C*drinking	*0.8680*	*0.4018*
7	LDL-C*hypertension	*0.7685*	*0.8919*
8	LDL-C*diabetes	0.7151	0.1260
9	LDL-C*heart disease	0.8480	0.9988
10	LDL-C*stroke	0.1101	0.3961
11	LDL-C*cancer	0.6451	0.6695
12	LDL-C*lung disease	0.6657	0.4847
13	LDL-C*memory disease	0.6225	0.4887
14	LDL-C*kidney disease	0.1251	0.1876
15	LDL-C*arthritis	0.4297	0.1671
16	LDL-C*asthma	0.4187	0.4779
17	LDL-C*liver disease	0.6929	0.7013
18	LDL-C*digestive disease	0.1048	0.4019
19	LDL-C*psychological disease	0.4605	0.3731

For peer review only

Supplementary Table S3 Associations between all-cause mortality and LDL-C#

	Total	Deaths (%)	Unadjusted		Adjusted*	
			HR (95%CI)	*P* value	HR (95%CI)	*P* value
Men						
Q1	972	69 (7.10)	1	-	1	-
Q2	999	58 (5.81)	0.806(0.568~1.142)	0.2251	0.921(0.582~1.457)	0.7236
Q3	979	45 (4.60)	0.642(0.441~0.934)	0.0205	0.762(0.472~1.231)	0.2668
Q4	992	35 (3.53)	0.491(0.327~0.737)	0.0006	0.566(0.337~0.951)	0.0315
Q5	982	41 (4.18)	0.579(0.394~0.853)	0.0056	0.841(0.510~1.386)	0.4970
Women						
Q1	1106	44 (3.98)	1	-	1	-
Q2	1094	41 (3.75)	0.951(0.622~1.456)	0.8177	1.171(0.683~2.005)	0.5660
Q3	1094	27 (2.47)	0.622(0.385~1.005)	0.0523	0.648(0.350~1.198)	0.1664
Q4	1105	35 (3.17)	0.799(0.513~1.246)	0.3222	0.815(0.454~1.462)	0.4924
Q5	1099	41 (3.73)	0.934(0.610~1.430)	0.7538	0.910(0.519~1.596)	0.7424

#Participants who died during the first year were excluded.

*Adjusted for age, smoking, drinking, BMI, living alone, household income, educational level, rural residence, ADL disability, hand grip strength, HDL-C, triglyceride, hemoglobin, hypertension, HBS/diabetes, history of stroke, cancer, heart disease, lung disease, liver disease, kidney disease, digestive disease, asthma, arthritis, psychological problem and memory problem.

For peer review only

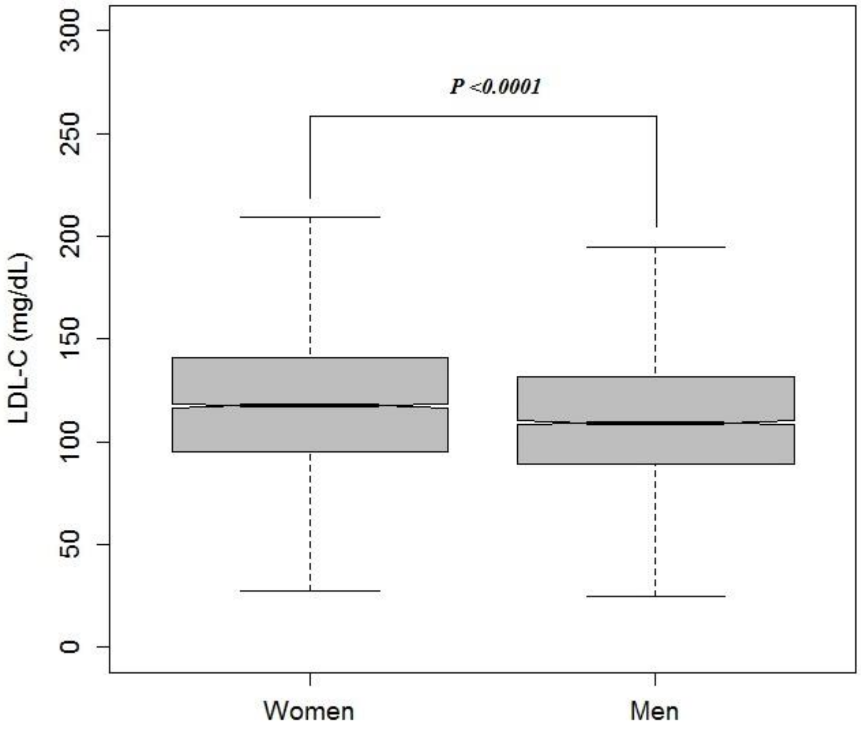

Supplementary Figure S1 The box-plot of plasma LDL-C levels in middle-aged and elderly Chinese men and women.

For peer review only

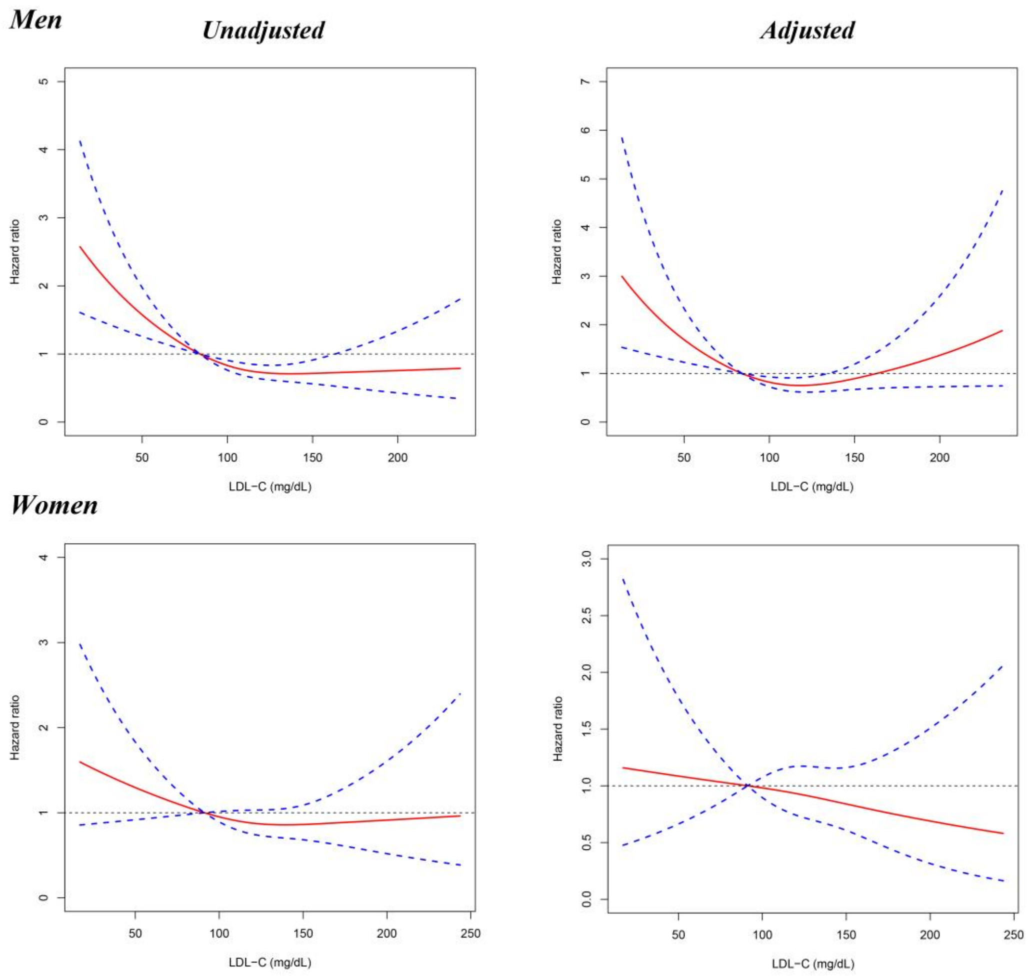

Supplementary Figure S2 Results from restricted cubic splines for the association between LDL-C and 4-year all-cause mortality in men and women (excluding participants who died during the first year). The multivariable models were adjusted for age, smoking, drinking, BMI, living alone, household income, educational level, rural residence, ADL disability, hand grip strength, HDL-C, triglyceride, hemoglobin, hypertension, HBS/diabetes, history of stroke, cancer, heart disease, lung disease, liver disease, kidney disease, digestive disease, asthma, arthritis, psychological problem and memory problem.

STROBE 2007 (v4) Statement—Checklist of items that should be included in reports of *cohort studies*

Section/Topic	Item #	Recommendation	Reported on page #
**Title and abstract**	1	(*a*) Indicate the study's design with a commonly used term in the title or the abstract	2
		(*b*) Provide in the abstract an informative and balanced summary of what was done and what was found	2
**Introduction**			
Background/rationale	2	Explain the scientific background and rationale for the investigation being reported	3
Objectives	3	State specific objectives, including any prespecified hypotheses	4
**Methods**			
Study design	4	Present key elements of study design early in the paper	4
Setting	5	Describe the setting, locations, and relevant dates, including periods of recruitment, exposure, follow-up, and data collection	4,5
Participants	6	(*a*) Give the eligibility criteria, and the sources and methods of selection of participants. Describe methods of follow-up	4,5
		(*b*) For matched studies, give matching criteria and number of exposed and unexposed	Not applicable
Variables	7	Clearly define all outcomes, exposures, predictors, potential confounders, and effect modifiers. Give diagnostic criteria, if applicable	4,5
Data sources/ measurement	8*	For each variable of interest, give sources of data and details of methods of assessment (measurement). Describe comparability of assessment methods if there is more than one group	*4,5*
Bias	9	Describe any efforts to address potential sources of bias	4,7,8,9
Study size	10	Explain how the study size was arrived at	4
Quantitative variables	11	Explain how quantitative variables were handled in the analyses. If applicable, describe which groupings were chosen and why	5
Statistical methods	12	(*a*) Describe all statistical methods, including those used to control for confounding	5,6
		(*b*) Describe any methods used to examine subgroups and interactions	5,6,7
		(*c*) Explain how missing data were addressed	4,5
		(*d*) If applicable, explain how loss to follow-up was addressed	5
		(*e*) Describe any sensitivity analyses	4,7,8,9
**Results**			

For peer review only

Participants	13*	(a) Report numbers of individuals at each stage of study—eg numbers potentially eligible, examined for eligibility, confirmed eligible, included in the study, completing follow-up, and analysed	4,5
		(b) Give reasons for non-participation at each stage	4,5
		(c) Consider use of a flow diagram	4
Descriptive data	14*	(a) Give characteristics of study participants (eg demographic, clinical, social) and information on exposures and potential confounders	6
		(b) Indicate number of participants with missing data for each variable of interest	4
		(c) Summarise follow-up time (eg, average and total amount)	5
Outcome data	15*	Report numbers of outcome events or summary measures over time	7
Main results	16	(*a*) Give unadjusted estimates and, if applicable, confounder-adjusted estimates and their precision (eg, 95% confidence interval). Make clear which confounders were adjusted for and why they were included	6,7
		(*b*) Report category boundaries when continuous variables were categorized	5,6,7
		(*c*) If relevant, consider translating estimates of relative risk into absolute risk for a meaningful time period	Not applicable
Other analyses	17	Report other analyses done—eg analyses of subgroups and interactions, and sensitivity analyses	6,7,8,9
**Discussion**			
Key results	18	Summarise key results with reference to study objectives	8
**Limitations**			
Interpretation	20	Give a cautious overall interpretation of results considering objectives, limitations, multiplicity of analyses, results from similar studies, and other relevant evidence	8,9,10
Generalisability	21	Discuss the generalisability (external validity) of the study results	8,9,10
**Other information**			
Funding	22	Give the source of funding and the role of the funders for the present study and, if applicable, for the original study on which the present article is based	11

*Give information separately for cases and controls in case-control studies and, if applicable, for exposed and unexposed groups in cohort and cross-sectional studies.

Note: An Explanation and Elaboration article discusses each checklist item and gives methodological background and published examples of transparent reporting. The STROBE checklist is best used in conjunction with this article (freely available on the Web sites of PLoS Medicine at http://www.plosmedicine.org/, Annals of Internal Medicine at http://www.annals.org/, and Epidemiology at http://www.epidem.com/). Information on the STROBE Initiative is available at www.strobe-statement.org.

For peer review only
